# Type I Interferon Induced Epigenetic Regulation of Macrophages Suppresses Innate and Adaptive Immunity in Acute Respiratory Viral Infection

**DOI:** 10.1371/journal.ppat.1005338

**Published:** 2015-12-28

**Authors:** Danielle N. Kroetz, Ronald M. Allen, Matthew A. Schaller, Cleyton Cavallaro, Toshihiro Ito, Steven L. Kunkel

**Affiliations:** 1 Department of Pathology, University of Michigan, Ann Arbor, Michigan, United States of America; 2 Department of Immunology, Nara Medical University, Nara, Japan; St. Jude Children's Research Hospital, UNITED STATES

## Abstract

Influenza A virus (IAV) is an airborne pathogen that causes significant morbidity and mortality each year. Macrophages (Mϕ) are the first immune population to encounter IAV virions in the lungs and are required to control infection. In the present study, we explored the mechanism by which cytokine signaling regulates the phenotype and function of Mϕ via epigenetic modification of chromatin. We have found that type I interferon (IFN-I) potently upregulates the lysine methyltransferase *Setdb2* in murine and human Mϕ, and in turn Setdb2 regulates Mϕ-mediated immunity in response to IAV. The induction of *Setdb2* by IFN-I was significantly impaired upon inhibition of the JAK-STAT signaling cascade, and chromatin immunoprecipitation revealed that both STAT1 and interferon regulatory factor 7 bind upstream of the transcription start site to induce expression. The generation of *Setdb2* ^*LacZ*^ reporter mice revealed that IAV infection results in systemic upregulation of *Setdb2* in myeloid cells. In the lungs, alveolar Mϕ expressed the highest level of *Setdb2*, with greater than 70% *lacZ* positive on day 4 post-infection. Silencing Setdb2 activity in Mϕ *in vivo* enhanced survival in lethal IAV infection. Enhanced host protection correlated with an amplified antiviral response and less obstruction to the airways. By tri-methylating H3K9, Setdb2 silenced the transcription of *Mx1* and *Isg15*, antiviral effectors that inhibit IAV replication. Accordingly, a reduced viral load in knockout mice on day 8 post-infection was linked to elevated *Isg15* and *Mx1* transcript in the lungs. In addition, Setdb2 suppressed the expression of a large number of other genes with proinflammatory or immunomodulatory function. This included *Ccl2*, a chemokine that signals through CCR2 to regulate monocyte recruitment to infectious sites. Consistently, knockout mice produced more CCL2 upon IAV infection and this correlated with a 2-fold increase in the number of inflammatory monocytes and alveolar Mϕ in the lungs. Finally, Setdb2 expression by Mϕ suppressed IL-2, IL-10, and IFN-γ production by CD4^+^ T cells *in vitro*, as well as proliferation in IAV-infected lungs. Collectively, these findings identify Setdb2 as a novel regulator of the immune system in acute respiratory viral infection.

## Author Summary

IAV causes seasonal epidemics that result in significant morbidity and mortality annually. Less frequently, novel viral strains emerge and are responsible for much larger outbreaks around the globe. In the last pandemic in 2009, an estimated 300,000 people died from IAV infection or secondary complications. Since the virus rapidly evolves, a new vaccine must be developed each year. Since vaccine effectiveness can be highly variable, identifying other therapeutic targets is appealing for the treatment of severe disease in high-risk individuals such as young children, the elderly, and immunocompromised individuals. In this study, we found that the protein Setdb2 regulates the immune response to IAV via an epigenetic mechanism in Mϕ. Inhibition of Setdb2 activity was beneficial for host protection due to an amplified antiviral response, which correlated with accelerated viral clearance and less damage to the lungs. Therefore, targeting Setdb2 may be a powerful therapeutic strategy for treating severe pulmonary disease caused by IAV and potentially other viral pathogens that trigger robust IFN-I production.

## Introduction

IAV is an airborne pathogen that is responsible for significant mortality in humans [1]. Infection with seasonal strains of IAV is typically limited to the upper respiratory tract and causes mild to moderately severe respiratory disease. In contrast, highly pathogenic strains of IAV can spread to distal airways and alveolar spaces causing pneumonia that can be lethal. Alveolar macrophages (Mϕ) are the first immune population to encounter IAV virions in the lungs and are required for host protection [2–5]. Following activation, alveolar Mϕ become highly phagocytic and are a major source of proinflammatory cytokines, including type I interferon (IFN-I) [6,7]. Viral detection by pattern recognition receptors (PRRs) initiates a signaling cascade that activates interferon regulatory factor (IRF) 3 and IRF7, transcription factors involved in the initiation and amplification of the IFN-I response [8,9]. IFN-I binds to the IFN-α receptor (IFNAR) to induce the transcription of more than 300 IFN-stimulated genes (ISGs) with antiviral and immunomodulatory functions [10]. However, the production of IFN-I and other proinflammatory cytokines must be tightly regulated to avoid respiratory failure. Cytokine-induced lung injury, rather than uncontrolled viral replication, is the most common cause of severe morbidity and mortality in individuals exposed to highly pathogenic strains of IAV [11–13].

Several other functions of resident and recruited Mϕ in infection caused by respiratory pathogens have been described. Early production of chemokines by alveolar Mϕ promotes the infiltration of inflammatory cells to the site of infection [14]. Additionally, Mϕ directly initiate adaptive immune responses during infection. It has been shown that alveolar Mϕ rapidly transport antigen to draining lymph nodes in *Streptococcus pneumoniae* infection [15]. Within the lungs, Mϕ present antigen and activate virus-specific T cells [16]. Expression of the Notch ligand Delta-like 1 by Mϕ regulates the production of the antiviral cytokine IFN-γ by CD4^+^ and CD8^+^ T cells in IAV infection [5]. Mϕ further enhance T cell-mediated immunity by undergoing apoptosis, resulting in cross-presentation of antigen to cytotoxic CD8^+^ T cells by DCs [17,18]. Finally, Mϕ play a pivotal role in the resolution of infection and restoration of an anti-inflammatory environment in the lungs. Internalization of residual infected-apoptotic cells and cellular debris by Mϕ inhibits viral dissemination and tissue damage by dampening inflammation and maintaining lung function [19–21].

Epigenetic modifications control gene transcription by altering residues in histone tails of chromatin. It has been shown that specific chromatin-modifying enzymes influence the phenotype and function of Mϕ [22–25]. The SET (Su(var)3-9, Enhancer-of-zeste, Trithorax)-domain superfamily consists of histone-modifying enzymes that transfer a methyl group from S-adenosyl-L-methionine to specific lysine residues in histone tails to either activate or block transcription [26]. Setdb2 (SET-domain bifurcated 2) tri-methylates lysine 9 of histone H3 (H3K9me3) to silence gene expression and was first implicated in the induction of B cell chronic lymphocytic leukemia [27]. Consistent with a recent publication by Schliehe *et al*., we demonstrate that cytokine-dependent signal transduction following IFN-I treatment upregulates Setdb2 in myeloid cells in a STAT1- and IRF7-dependent manner. However, despite this overlapping observation, we uncovered a role for Setdb2 in the regulation of the innate and adaptive immune system in primary IAV infection. Generation of mice lacking Setdb2 specifically in Mϕ revealed that Setdb2 controlled the recruitment of inflammatory monocytes to infected lungs and suppressed the expression of a large number of antiviral genes. Setdb2 expression by Mϕ also influenced cytokine production by CD4^+^ T cells, as well as proliferation of both CD4^+^ and CD8^+^ T cells in infected lungs. These results highlight the impact of histone modification in dictating the severity of infection and may represent a potential therapeutic target for controlling pulmonary infection and other diseases associated with IFN-I activity.

## Results

### IFN-I upregulates the lysine methyltransferase *Setdb2* in murine and human Mϕ

**Fig 1:**
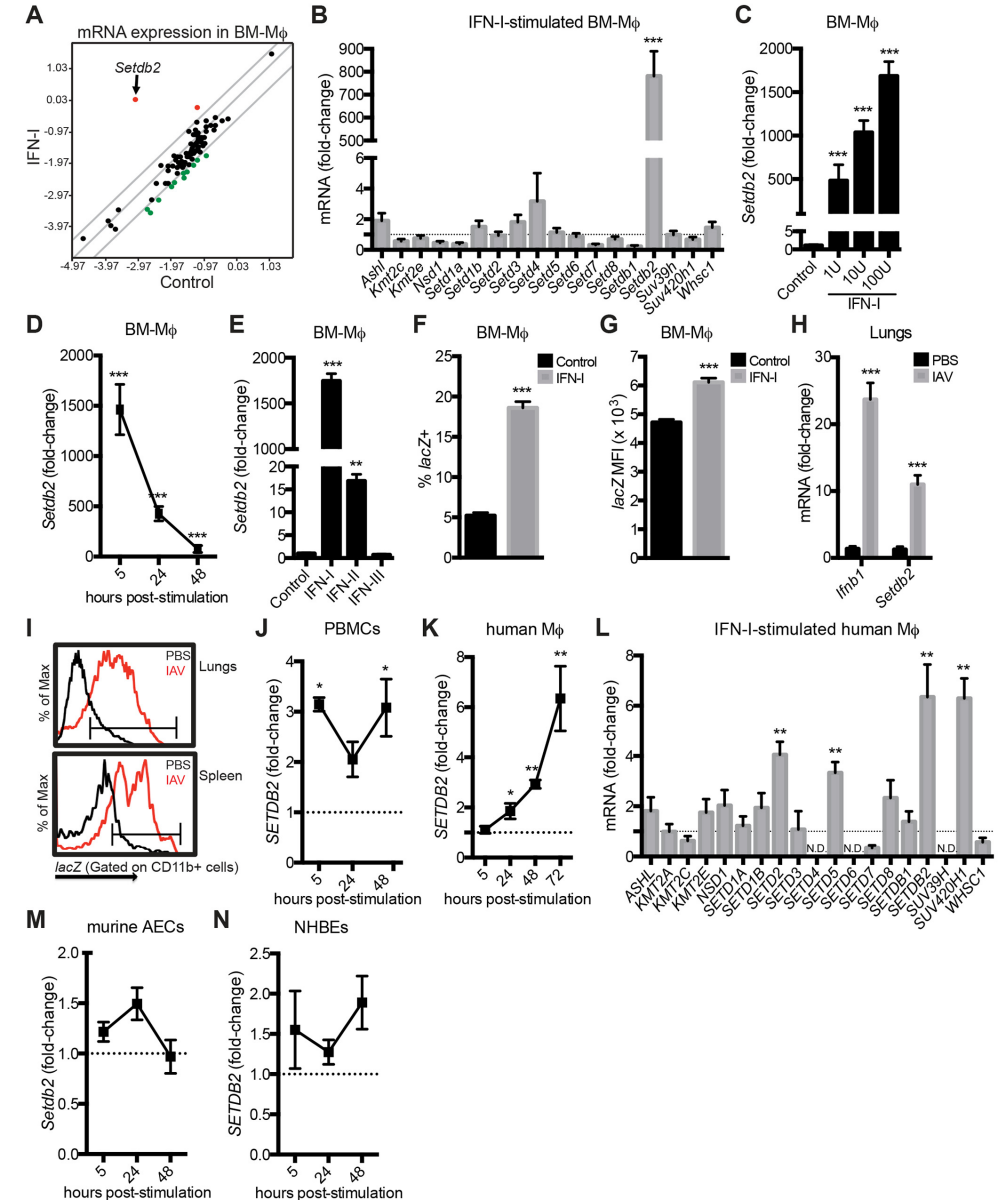
IFN-I upregulates the lysine methyltransferase *Setdb2* in Mϕ. **(A)** Comparison of chromatin-modifying enzymes in unstimulated (control) and IFN-I-stimulated BM-Mϕ by RT-PCR. Values on scatter plot represent log_10_ (Δ^Ct^) at 24 hours post-stimulation. The gray lines represent a 4-fold change in gene expression; upregulated genes (red dots), downregulated genes (green dots). **(B)** RT-PCR of SET-domain superfamily genes in BM-Mϕ treated with a vehicle control (dotted line) or IFN-I for 24 hours. **(C-E)** RT-PCR of *Setdb2* by BM-Mϕ stimulated with 1, 10, and 100 units/mL of IFN-I for 5 hours **(C)**, stimulated with IFN-I for 5, 24, and 48 hours **(D)**, and stimulated with IFN-I, IFN-II, and IFN-III for 5 hours **(E)**. **(B-E)** Data are mean ± SEM relative to unstimulated BM-Mϕ, n = 3–4 independent experiments. **(F, G)** *lacZ* expression **(F)** and MFI **(G)** in BM-Mϕ isolated from *Setdb2* ^*LacZ*^ reporter mice was determined by flow cytometry 24 hours post-IFN-I stimulation. Data are mean ± SEM gating on CD11b^+^ cells, n = 3 independent experiments. **(H, I)** C57BL/6 **(H)** and *Setdb2* ^*LacZ*^ reporter **(I)** mice were inoculated with PBS or IAV (1 x 10^4^ PFU). **(H)** RT-PCR of *Ifnb1* and *Setdb2* in IAV-infected lungs on day 4 post-infection. Data represent fold-change relative to uninfected lungs; data are mean ± SEM, n = 8–12 mice. **(I)** *lacZ* expression by CD11b^+^ cells in the lungs and spleen was determined by flow cytometry; PBS (black), IAV (red). Histograms are a representative example from 3 independent experiments (n = 8–12 mice). **(J, K)** RT-PCR of *Setdb2* in human PBMCs **(J)** and M-CSF-skewed Mϕ **(K)** stimulated with IFN-I over the indicated time course. **(L)** RT-PCR of SET-domain superfamily genes in human Mɸtreated with a vehicle control (dotted line) or IFN-I for 72 hours. **(M, N)** RT-PCR of *Setdb2* in murine AECs **(M)** and NHBEs **(N)** stimulated with IFN-I for the indicated time course. **(J-N)** Data are mean ± SEM relative to unstimulated cells (dotted line), n = 3–5 independent experiments. N.D.; not detected. *p<0.05; **p<0.01; ***p<0.001.

Specific cytokines can induce the expression of histone-modifying enzymes, which, in turn, regulate the transcription of target genes in a variety of immune responses [23,24]. Since IFN-I is rapidly produced following infection with a number of viral pathogens, we asked if IFN-I induced the expression of histone-modifying enzymes in bone marrow-derived Mϕ (BM-Mϕ). Notably, the lysine methyltransferase *Setdb2* was upregulated by more than 700-fold relative to unstimulated BM-Mϕ (1.0 ± 0.17 vs. 781.0 ± 108.2; *p*<0.001) at 24 hours post-stimulation (Fig 1A). IFN-I-dependent induction was specific to *Setdb2*, as related histone methyltransferases containing a SET-domain were unaltered following cytokine stimulation (Fig 1B). To determine if the induction of *Setdb2* occurred in a dose-dependent manner, BM-Mϕ were stimulated with increasing doses of cytokine. When normalized to unstimulated BM-Mϕ, a direct correlation between the concentration of IFN-I and *Setdb2* transcript was observed (Fig 1C). To characterize the kinetics of *Setdb2* expression, BM-Mϕ were treated with IFN-I over a time course. *Setdb2* transcription peaked at 5 hours post-stimulation, began declining by 24 hours, and returned to baseline levels by 48 hours (Fig 1D). We next examined whether cytokines related to IFN-I could upregulate *Setdb2*. BM-Mϕ were treated with IFN-γ (type II IFN) or IFN-λ (type III IFN) in parallel with IFN-I. While IFN-γ upregulated *Setdb2* relative to unstimulated cells (16.8 ± 1.48 vs. 1.0 ± 0.33; *p*<0.001), it was significantly less potent than IFN-I (Fig 1E).

To further characterize Setdb2 expression *in vitro* and *in vivo*, we generated *Setdb2* ^*LacZ*^ reporter mice. To measure the degree of transcription from the *Setdb2* promoter, a gene trap vector was used to incorporate the *E*. *coli* gene *lacZ*, which encodes β-galactosidase, into recombinant DNA to generate *lacZ* fusion transcripts as previously described [28]. BM-Mϕ from reporter mice were treated with a vehicle control or IFN-I and β-galactosidase activity was measured by flow cytometry. At 24 hours post-stimulation, 5% of control cells expressed *lacZ*. IFN-I treatment increased *Setdb2* expression, with more than 20% of BM-Mϕ *lacZ* positive (5.17 ± 0.48% vs. 22.3 ± 1.32%; *p*<0.001) (Fig 1F). Enhanced *lacZ* expression following IFN-I treatment correlated with an increase in mean fluorescent intensity (MFI) (4921 ± 35.2 vs. 6464 ± 52.3; *p*<0.001) (Fig 1G).

Since exposure to IAV triggers robust IFN-I production, we next characterized *Setdb2* expression in a murine model of infection. On day 4 post-infection, enhanced expression of *Ifnb1* correlated with a 11-fold increase in *Setdb2* transcript when normalized to uninfected lungs (11.0 ± 1.38 vs. 1.0 ± 0.43; *p*<0.001) (Fig 1H). To confirm these data, *Setdb2* ^*LacZ*^ reporter mice were inoculated with PBS or IAV. In naïve animals, less than 10% of CD11b^+^ cells in the lungs were *lacZ* positive. IAV infection enhanced the percentage of CD11b^+^ cells expressing *lacZ* in the lungs (7.25 ± 0.14% vs. 30.8 ± 2.25%; *p*<0.001) (Fig 1I). In addition, *Setdb2* was upregulated in the spleen following infection (7.38 ± 0.19% vs. 25.9 ± 2.37%; *p*<0.001) (Fig 1I). Enhanced *lacZ* expression by CD11b^+^ cells in IAV infection was accompanied by a significant shift in MFI in the lungs and spleen, respectively (2095 ± 41.3 vs. 2882 ± 29.9; *p*<0.001, 3576 ± 39.1 vs. 5280 ± 122.3; *p*<0.001).

These results prompted us to characterize *SETDB2* expression in human cells. Peripheral blood mononuclear cells (PBMCs) were isolated from healthy donors and stimulated with IFN-I. When normalized to unstimulated PBMCs, IFN-I treatment resulted in a 3-fold increase in *SETDB2* at 5 and 48 hours post-stimulation (Fig 1J). Since we only observed a slight increase in *SETDB2* in PBMCs, CD14^+^ monocytes were skewed toward a Mϕ phenotype and stimulated with IFN-I. Initially, IFN-I did not induce *SETDB2* expression in human Mϕ. However, a slight increase was observed by 24 hours and *SETDB2* expression continued to increase through 72 hours post-stimulation (Fig 1K). In contrast to murine BM-Mϕ, IFN-I upregulated *SETD2*, *SETD5*, and *SUV420* in human Mϕ at 72 hours post-stimulation (Fig 1L). Despite being the primary target for IAV, *Setdb2* was not induced in murine airway epithelial cells (AECs) or normal human bronchial epithelial cells (NHBEs) treated with IFN-I (Fig 1M and 1N).

### *Setdb2* expression by Mϕ is dependent on JAK-STAT pathway and IRF7

**Fig 2:**
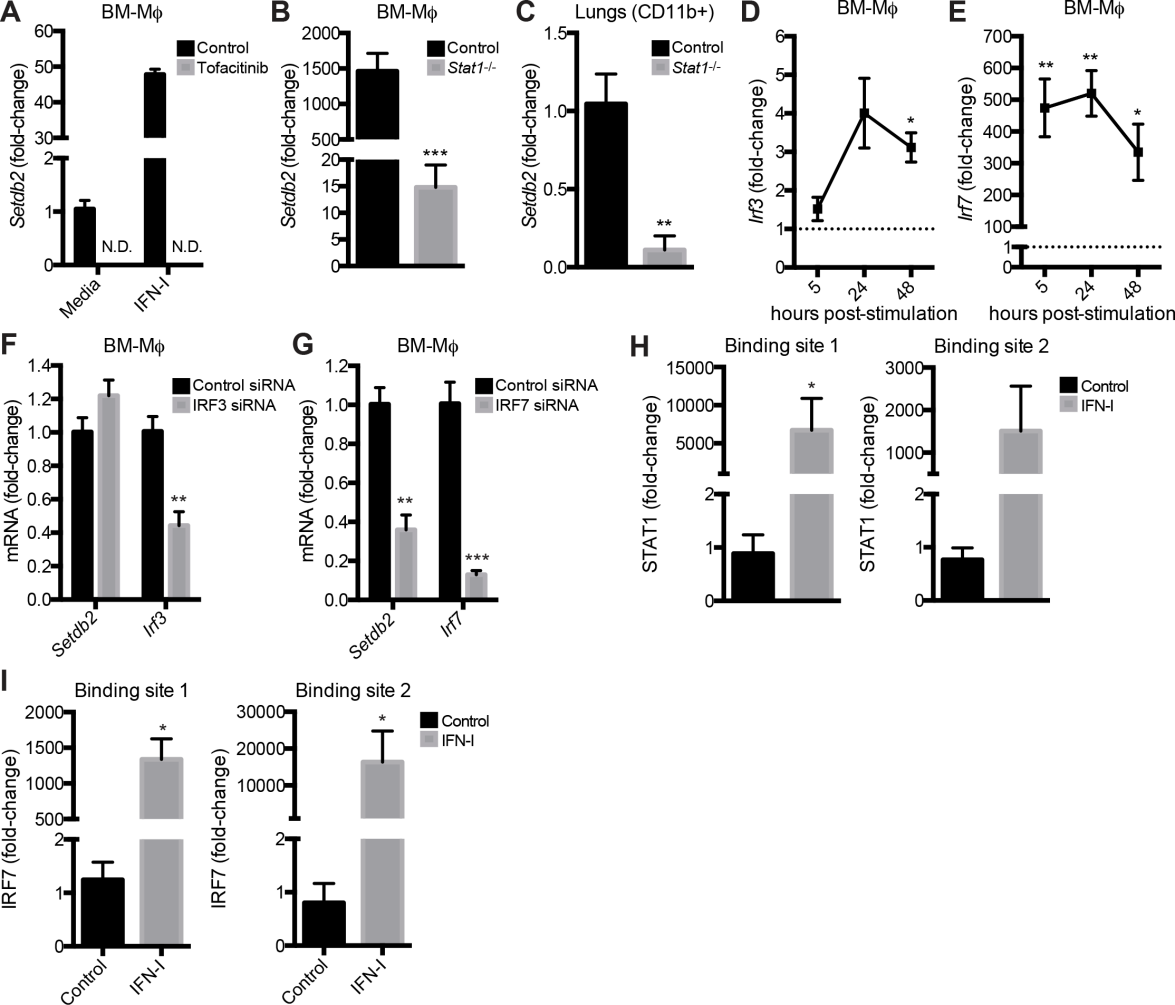
*Setdb2* expression by Mϕ is dependent on JAK-STAT pathway and IRF7. **(A-C)** RT-PCR of *Setdb2* in BM-Mϕ **(A, B)** and CD11b^+^ cells **(C)** from IAV-infected lungs. **(A)** Untreated and IFN-I-stimulated BM-Mϕ were treated with a vehicle control or the JAK inhibitor tofacitinib at the time of stimulation. **(B)** Control and *Stat1* ^-/-^ BM-Mϕ were stimulated with media or IFN-I for 5 hours. **(A, B)** Data are mean ± SEM relative to unstimulated cells; n = 3 independent experiments. **(C)** CD11b^+^ cells were isolated from the lungs of control and *Stat1* ^-/-^ mice inoculated with IAV (1 x 10^4^ PFU). Data are mean ± SEM relative to control cells on day 4 post-infection; n = 8–12 mice. **(D, E)** RT-PCR of *Irf3* **(D)** and *Irf7* **(E)** in control BM-Mϕ stimulated with IFN-I for the indicated time course. Data are mean ± SEM relative to unstimulated cells; n = 2–3 independent experiments **(F, G)** RT-PCR of *Setdb2* and *Irf3*/*Irf7* in BM-Mϕ treated with control, IRF3, or IRF7 siRNA and stimulated with IFN-I for 24 hours. Data are mean ± SEM relative to cells treated with control siRNA; n = 3 independent experiments. **(H, I)** ChIP analysis of STAT1 **(H)** and IRF7 **(I)** binding in the *Setdb2* promoter in control and IFN-I-stimulated BM- Mϕ after 24 hours. Data are mean ± SEM relative to IgG control; n = 2–3 independent experiments. N.D.; not detected. *p<0.05; **p<0.01; ***p<0.001.

IFN-I signals through the JAK-STAT signaling pathway to promote the transcription of ISGs involved in antiviral immunity. This prompted us to explore the signaling pathway regulating *Setdb2* transcription. We initially tested if IFN-I-dependent induction of *Setdb2* was dependent on the JAK-STAT pathway using the JAK inhibitor tofacitinib [29]. In comparison to control BM-Mϕ, *Setdb2* transcript was undetected in both unstimulated and IFN-I-stimulated BM-Mϕ treated with tofacitinib (Fig 2A). Next, we examined *Setdb2* expression in *Stat1* ^-/-^ mice since IFN-I signals predominantly through a STAT1-STAT2 heterodimer. Stimulation of wild-type BM-Mϕ with IFN-I resulted in nearly a 1500-fold increase *Setdb2* transcription (1463 ± 250; *p*<0.001). A deficiency in *Stat1* significantly dampened the induction of *Setdb2*, with less than a 15-fold induction relative to unstimulated BM-Mϕ (14.8 ± 4.20; *p*<0.001) (Fig 2B). In addition, *Setdb2* expression was impaired in the lungs of *Stat1* ^-/-^ mice infected with IAV. On day 4 post-infection, a 90% reduction in *Setdb2* transcript was observed in CD11b^+^ cells from *Stat1* ^-/-^ lungs relative to control lungs (Fig 2C).

IRF3 and IRF7 are critical transcription factors involved in IFN-I production and the induction of ISGs [30,31]. Whereas IRF3 is constitutively expressed at low levels and initiates IFN-I production after viral detection, IRF7 is an ISG that is expressed at high levels in infection and is the master regulator of the IFN-I response. Consistent with published data, stimulation of BM-Mϕ with IFN-I resulted in a dramatic upregulation of *Irf7*, but not *Irf3* (Fig 2D and 2E). To determine if *Setdb2* expression was dependent on either transcription factor, BM-Mϕ were transfected with control, IRF3, or IRF7 siRNA and stimulated with IFN-I. Silencing of IRF7, but not IRF3, resulted in a significant reduction in *Setdb2* in comparison to control cells (0.44 ± 0.08 vs. 1.0 ± 0.09; *p*<0.01). Transcription of *Irf3* and *Irf7* was diminished by at least 60% and 80%, respectively, when treated with respective siRNA indicating the knockdown was successful (Fig 2F and 2G).

Since *Setdb2* expression was diminished in BM-Mϕ deficient in *Stat1* or treated with IRF7 siRNA, we performed ChIP to determine if these transcription factors regulated expression by binding to the *Setbd2* promoter. Using published binding site sequences, we identified STAT1 and IRF7 binding sites upstream of the *Setdb2* transcription start site. Prior to cytokine stimulation, STAT1 and IRF7 were absent in the *Setdb2* promoter (Fig 2H and 2I). However, a dramatic increase in STAT1 and IRF7 binding was observed in BM-Mϕ stimulated with IFN-I (Fig 2H and 2I).

### *Setdb2* is systemically upregulated in myeloid cells in IAV infection

**Fig 3:**
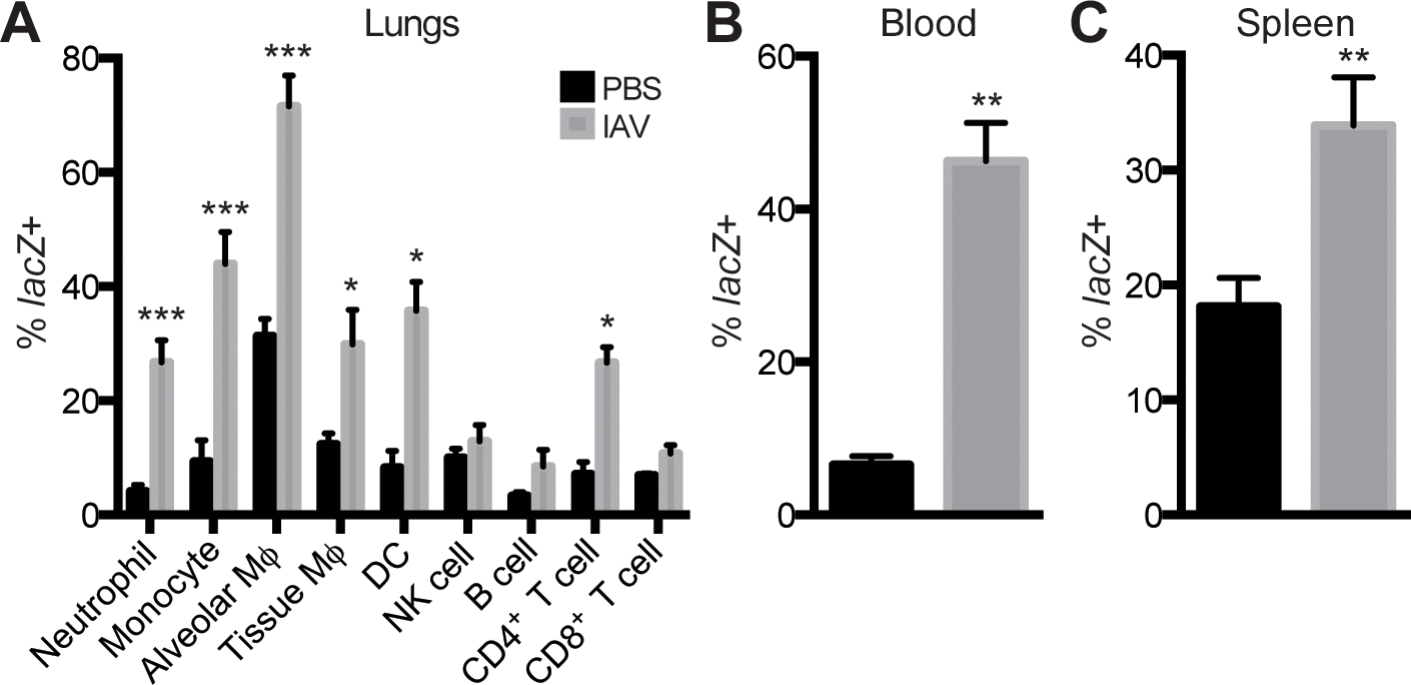
*Setdb2* is systemically upregulapted in a murine model of IAV infection. *Setdb2* ^*LacZ*^ reporter mice were inoculated with PBS or IAV (1 x 10^4^ PFU) to determine the proportion of inflammatory populations expressing *lacZ* in the lungs **(A)**, blood **(B)**, and spleen **(C)** on day 0 and 4 post-infection. Leukocytes were phenotypically characterized by the following surface markers: neutrophils (Ly6G^high^ CD11b^+^ CD11c^-^), inflammatory monocytes (Ly6C^high^ CD11b^+^ CD11c^-^ CCR2^+^), alveolar Mϕ (F4/80^+^ CD11c^+^ CD11b^low/-^ MHC-II^low/int^), tissue Mϕ (F4/80^+^ CD11b^+^ CD11c^-^ MHC-II^high^), conventional DCs (MHC-II^high^ CD11b^+^ CD11c^+^), NK cells (NK1.1^+^ CD3^-^), B cells (B220^+^ CD4^-^ CD8^-^), CD4^+^ T cells (CD4^+^ CD3^+^), and CD8^+^ T cells (CD8^+^ CD3^+^). Data are mean ± SEM (n = 6–12 mice) from 2–3 independent experiments. *p<0.05; **p<0.01; ***p<0.001.

Since IAV infection results in the influx of inflammatory cells to the lungs, we further characterized *Setdb2* expression using *Setdb2* ^*LacZ*^ reporter mice. In naïve lungs, alveolar Mϕ expressed the highest level of *Setdb2*, with 30% of the population *lacZ* positive. Less than 10% of other myeloid and lymphoid cellular populations expressed *lacZ* in steady-state conditions (Fig 3A). IAV infection resulted in upregulation of *Setdb2* in multiple cellular populations. Similar to uninfected lungs, alveolar Mϕ were the predominant population expressing *Setdb2* in IAV infection. On day 4 post-infection, the proportion of alveolar Mϕ expressing Setdb2 nearly doubled, with greater than 70% expressing *lacZ* (31.3 ± 1.74% vs. 74.6 ± 2.69%; *p*<0.001) (Fig 3A). In addition, infection enhanced *lacZ* expression in other myeloid populations, including inflammatory monocytes, tissue Mϕ, neutrophils, and DCs. In contrast, CD4^+^ T cells were the only lymphoid population to express more *lacZ* following infection. Comparable to naïve lungs, less than 10% of NK cells, CD8^+^ T cells, and B cells were *lacZ* positive in infected lungs (Fig 3A). IAV-dependent induction of *Setdb2* transcription was not limited to the lungs, as *lacZ* expression was significantly elevated in inflammatory monocytes in the blood (6.51 ± 1.16% vs. 46.3 ± 5.01%; *p*<0.01) and spleen (18.1 ± 2.48% vs. 33.9 ± 4.19%; *p*<0.01) (Fig 3B and 3C).

### *Setdb2* expression by specific myeloid populations controls the severity of IAV infection

**Fig 4:**
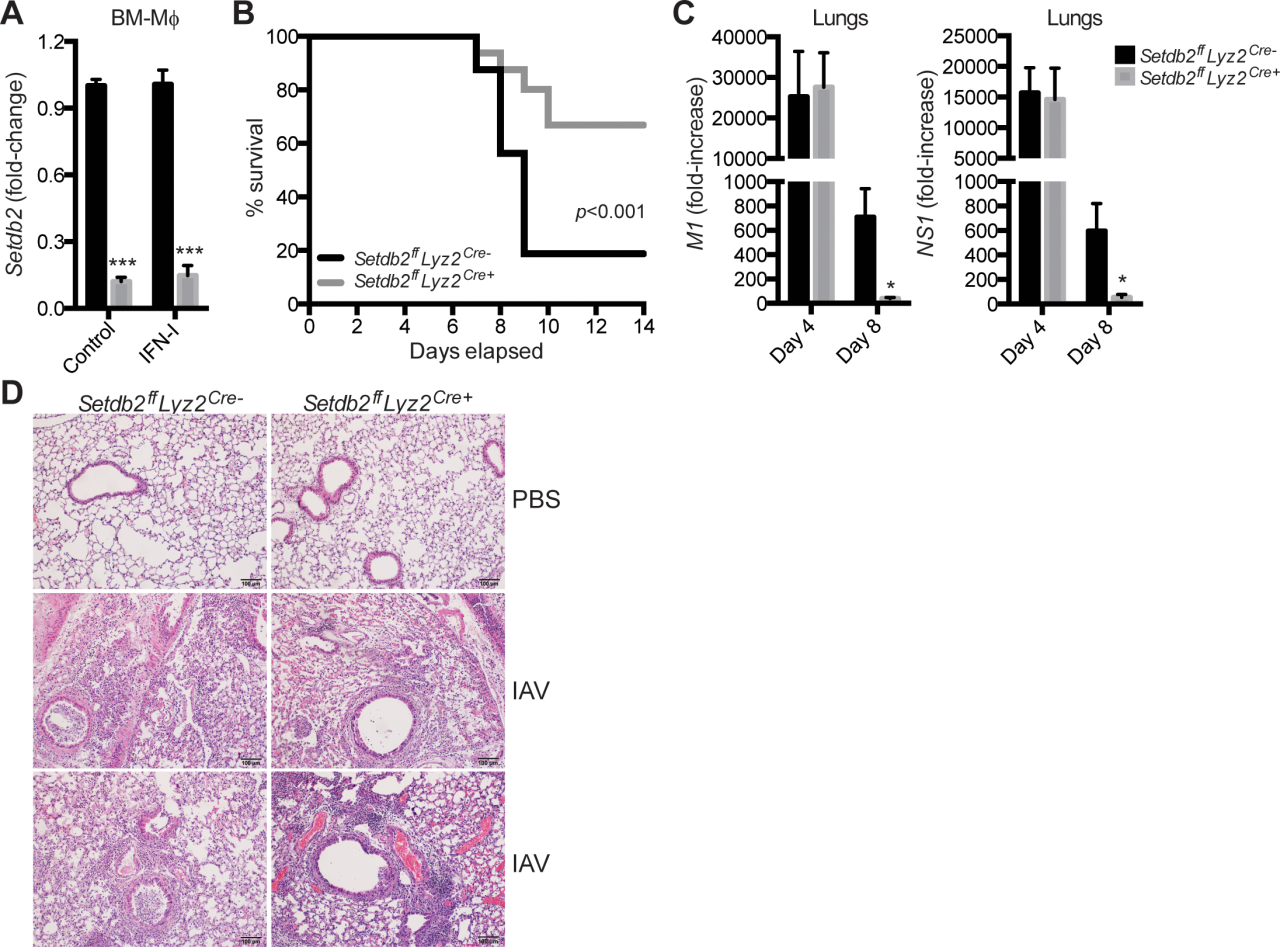
*Setdb2* expression by specific myeloid populations controls the severity of IAV infection. **(A)** RT-PCR of *Setdb2* in control and *Setdb2* ^-/-^ BM-Mϕ treated with a vehicle control or IFN-I for 5 hours. Data are mean ± SEM from 3 independent experiments. **(B)** Survival of control and *Setdb2* ^*ff*^ *Lyz2* ^*cre+*^ mice inoculated with a lethal dose of IAV (1 x 10^5^ PFU). **(C, D)** Control and *Setdb2* ^*ff*^ *Lyz2* ^*cre+*^ mice inoculated with a sublethal dose of IAV (1 x 10^4^ PFU). **(C)** RT-PCR of *M1* and *NS1* in infected lungs on day 4 and 8 post-infection. **(D)** Lung sections were stained with H&E on day 0 and 4 post-infection. Scale bar, 100 μm. **(B-D)** Data are from 3 independent experiments; n = 8–18 mice. *p<0.05; ***p<0.001.

To determine the role of Setdb2 in immunity during respiratory viral infection, we generated mice deficient for Setdb2 in myeloid cells with lysosomes (monocytes, Mϕ, and granulocytes) using the Cre-lox system. For validation, *Setdb2* transcription was measured in BM-Mϕ from *Setdb2* ^*ff*^ *Lyz2* ^*cre-*^ (control) and *Setdb2* ^*ff*^ *Lyz2* ^*cre+*^ (knockout) mice. Relative to control BM-Mϕ, a significant reduction in transcript was observed in *Setdb2* ^-/-^ BM-Mϕ treated with a vehicle control or IFN-I (Fig 4A). To examine if Setdb2 influenced the outcome of infection, mice were infected with a lethal dose of IAV and survival was monitored for two weeks. While both groups of mice began to succumb to infection on day 7, survival was enhanced in *Setdb2* ^*ff*^ *Lyz2* ^*cre+*^ mice. Whereas only 20% of control mice were alive by day 9, greater than 65% of knockout mice survived (Fig 4B). To determine viral load, we quantified the number of copies of IAV proteins in the lungs. While we observed a comparable fold-increase of non-structural protein 1 (*NS1*) and matrix protein 1 (*M1*) at day 4 post-infection, *Setdb2* ^*ff*^ *Lyz2* ^*cre+*^ lungs had a reduction in both viral proteins by day 8 post-infection (Fig 4C).

Since tissue damage is often the cause of morbidity and mortality in IAV infection, we next examined lung histology in naïve and infected mice. Prior to infection, control and *Setdb2* ^*ff*^ *Lyz2* ^*cre+*^ lung histology was comparable (Fig 4D). Indicative of respiratory viral infection, the airways of control lungs were filled, likely with dead epithelial and inflammatory cells, cellular debris, and virus on day 4 post-infection. In contrast, *Setdb2* ^*ff*^ *Lyz2* ^*cre+*^ lungs had less obstruction to the airways. In addition, we observed dense clusters of lymphoid cells near blood vessels in knockout mice that were absent in wild-type lungs (Fig 4D).

### Setdb2 suppresses the expression of proinflammatory and antiviral genes

**Fig 5:**
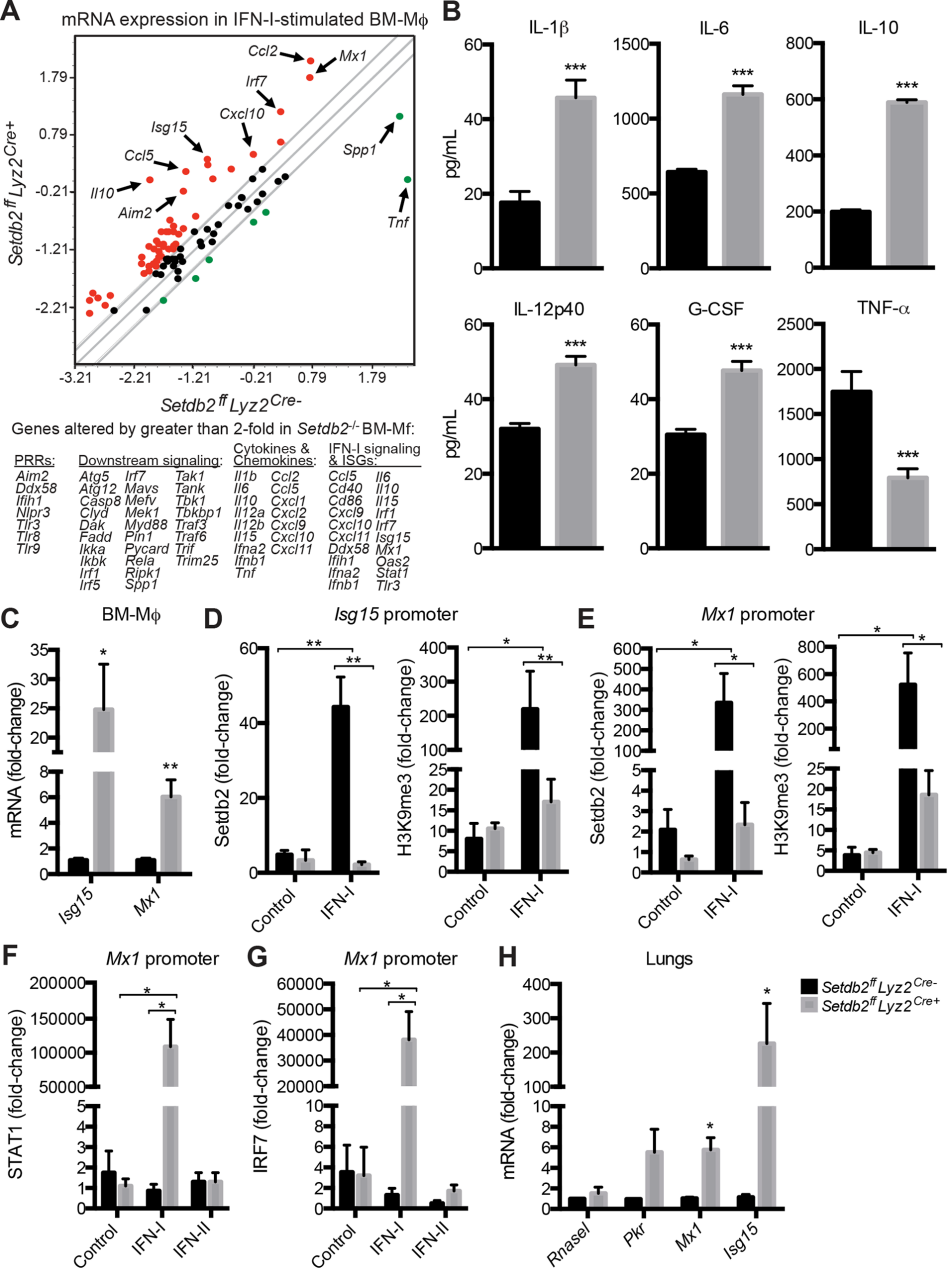
Setdb2 suppresses the expression of antiviral genes and cytokine production nu BM-Mϕ. Gene expression **(A, C)** and cytokine production **(B)** by control and *Setdb2* ^-/-^ BM-Mϕ stimulated with IFN-I for 24 hours. Data represents the mean ± SEM of 3 independent experiments. **(A)** Comparison of antiviral and proinflammatory genes by RT-PCR. Values on scatter plot represent log_10_ (Δ^Ct^). The gray lines represent a 2-fold change in gene expression; upregulated genes (red dots), downregulated genes (green dots). **(B)** Concentration of IL-1β, IL-6, IL-10, IL-12p40, G-GSF, and TNF-α in the supernatants of control and *Setdb2* ^-/-^ BM-Mϕ. **(C)** RT-PCR of *Isg15* and *Mx1*. Data were normalized to control BM-Mϕ. **(D-G)** Control and *Setdb2* ^-/-^ BM-Mϕ were treated with a vehicle control, IFN-I **(D-G)**, or IFN-II **(F, G)** for 24 hours. Data are mean ± SEM relative to IgG control; n = 2–3 experiments **(D, E)** ChIP analysis of Setdb2 binding and H3K9 tri-methylation in the *Isg15* **(D)** and *Mx1* **(E)** promoter. **(F, G)** ChIP analysis of STAT1 **(F)** and IRF7 **(G)** binding in the *Mx1* promoter. **(H)** Control and *Setdb2* ^*ff*^ *Lyz2* ^*cre+*^ mice inoculated with a sublethal dose of IAV (1 x 10^4^ PFU).**)** RT-PCR of *Rnasel*, *Pkr*, *Mx1*, and *Isg15* in infected lungs on day 4 post-infection. Data are mean ± SEM (n = 6–14 mice) from 2–3 independent experiments. *p<0.05; **p<0.01; ***p<0.001.

**Fig 6:**
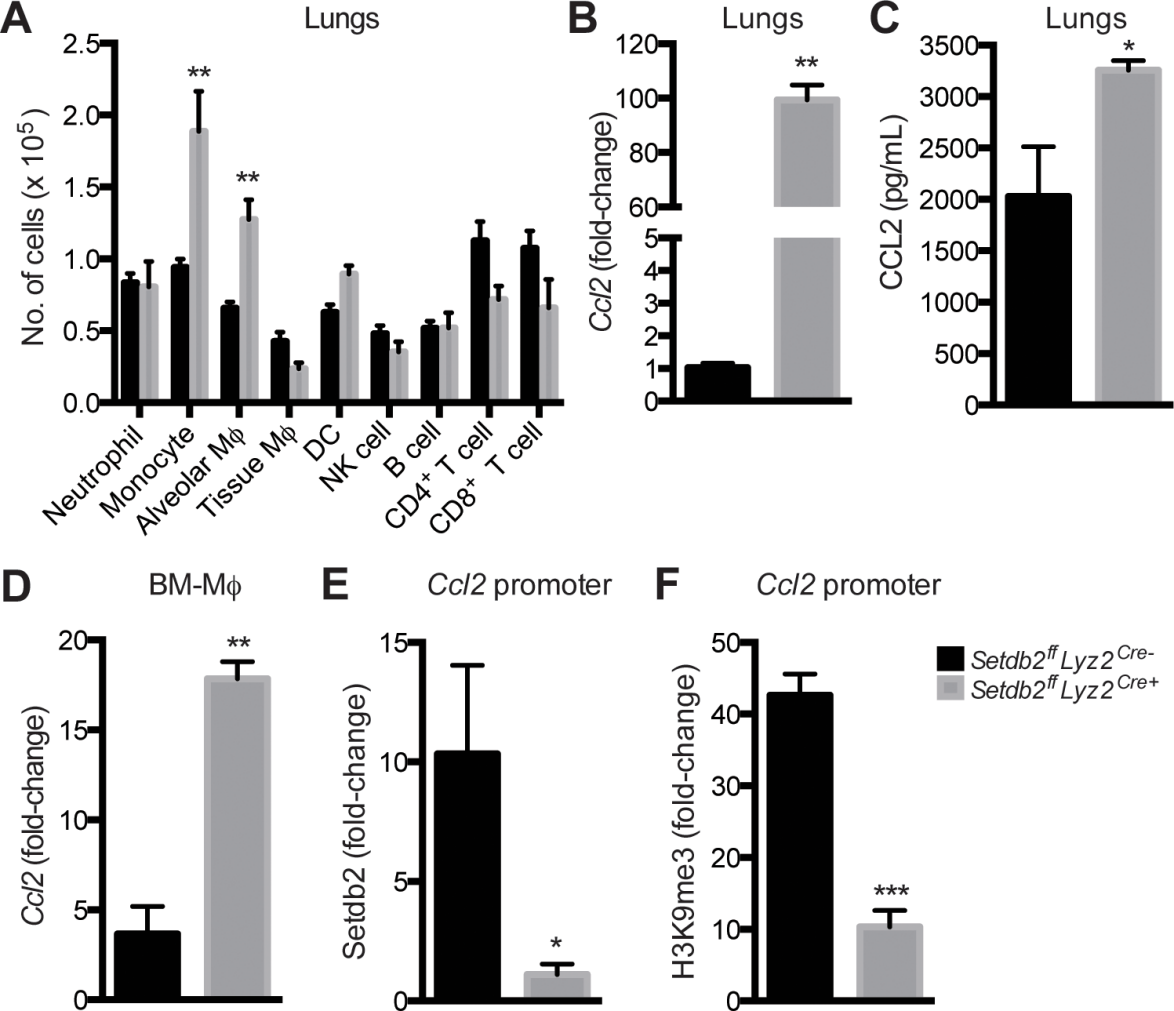
Setdb2 expression by Mϕ influences the influx of myeloid cells to the lungs following IAV infection. **(A-C)** Control and *Setdb2* ^*ff*^ *Lyz2* ^*cre+*^ mice were inoculated with IAV (1 x 10^4^ PFU) for 4 days. **(A)** The absolute number of inflammatory populations in IAV-infected lungs. Cells were surface stained for the following markers: neutrophils (Ly6G^high^ CD11b^+^ CD11c^-^), inflammatory monocytes (Ly6C^high^ CD11b^+^ CD11c^-^ CCR2^+^), alveolar Mϕ (F4/80^+^ CD11c^+^ CD11b^low/-^ MHC-II^low/int^), tissue Mϕ (F4/80^+^ CD11b^+^ CD11c^-^ MHC-II^high^), conventional DCs (MHC-II^high^ CD11b^+^ CD11c^+^), NK cells (NK1.1^+^ CD3^-^), B cells (B220^+^ CD4^-^ CD8^-^), CD4^+^ T cells (CD4^+^ CD3^+^), and CD8^+^ T cells (CD8^+^ CD3^+^). **(B)** RT-PCR of *Ccl2* in IAV-infected lungs. Values represent fold-change relative to control lungs. **(C)** CCL2 protein concentration in IAV-infected lungs. **(A-C)** Values represent mean ± SEM (n = 6–12 mice) from 2–3 independent experiments. **(D-F)** Control and *Setdb2* ^-/-^ BM-Mϕ were stimulated with IFN-I for 24 hours. **(D)** RT-PCR of *Ccl2* in BM-Mϕ Data are mean ± SEM relative to control BM-Mϕ, n = 3 independent experiments. **(E, F)** ChIP analysis of Setdb2 binding **(E)** and H3K9 tri-methylation **(F)** in the *Ccl2* promoter. Values represent fold-change relative to IgG controls. Values represent mean ± SEM from 2–3 independent experiments. *p<0.05; **p<0.01.

IAV sensing by the innate immune system results in robust production of IFN-I and other proinflammatory cytokines and chemokines. Since knockout mice controlled infection better than their wild-type counterparts, we postulated that Setbd2 dictates the severity of infection by regulating the transcription of antiviral genes. To identify potential target genes, we screened antiviral genes in control and *Setdb2* ^-/-^ BM-Mϕ stimulated with IFN-I using a PCR array. In the absence of *Setdb2*, the overall gene profile in BM- Mϕ was altered at 24 hours post-stimulation (Fig 5A; *p*<0.001). A 5.8- and 6.8-fold increase in *Ifna2* and *Ifnb1* transcript, respectively, was observed in *Setdb2* ^-/-^ BM-Mϕ. Notably, the CCR2 and CCR5 ligands *Ccl2* and *Ccl5*, respectively, were upregulated 23- and 29-fold in *Setdb2* ^-/-^ BM-Mϕ (Figs 5A and 6D). Additional chemokines and cytokines, including *Cxcl1*, *Cxcl2*, *Cxcl9*, *Cxcl10*, *Cxcl11*, *Il1b*, *Il6*, *Il10*, *Il12a*, *Il12b*, and *Il15* were upregulated by 2-fold or more in the absence of *Setdb2*. To confirm the cytokine transcription data, we measured the concentration of select cytokines in the supernatants of BM-Mϕ stimulated with IFN-I. Consistently, more IL-1β, IL-6, IL-10, IL-12p40, and G-CSF were detected in the absence of *Setdb2*. TNF-α transcript and protein was the only cytokine examined that was reduced in *Setdb2* ^-/-^ BM-Mϕ (Fig 5B).

IFN-I induces the expression of ISGs that inhibit viral replication and spreading [32,33]. Elevated *Ifna2* and *Ifnb1* in *Setdb2* ^-/-^ BM-Mϕ correlated with a 22.7- and 8.5-fold increase in *Isg15* and *Mx1* transcript, respectively (Fig 5C). Since Setdb2 silences gene expression, we performed ChIP to determine the presence of Setdb2 and H3K9me3 in the promoter region of both genes. In control BM-Mϕ, IFN-I stimulation resulted in a 40- and 300-fold increase in Setdb2 bound to the *Isg15* and *Mx1* promoters, respectively (Fig 5D and 5E). This correlated with high levels of H3K9me3 in the promoter region of both genes (Fig 5D and 5E). Relative to control BM-Mϕ a significant reduction in Setdb2 and H3K9me3 was observed in *Setdb2* ^-/-^ BM-Mϕ (Fig 5D and 5E). In addition to less Setdb2 and H3K9me3, enhanced *Mx1* expression in *Setdb2* ^-/-^ BM-Mϕ was associated with STAT1 and IRF7 bound to the promoter (Fig 5F and 5G). This was specific to IFN-I, as stimulation with IFN-II did not increase STAT1 or IRF7 binding in the *Mx1* promoter (Fig 5F and 5G). To determine if enhanced survival and a reduced viral load was associated with an enhanced antiviral response, RNA was isolated from the lungs on day 4 post-infection. Consistent with BM-Mϕ data, *Ifna2* (1.36 ± 0.27 vs. 33.1 ± 20.8; *p*<0.05) and *Ifnb1* (1.47 ± 0.36 vs. 488.9 ± 241.4; *p*<0.05) transcript was elevated in the lungs from *Setdb2* ^*ff*^ *Lyz2* ^*cre+*^ mice. This correlated with a 5- and 200-fold increase in *Mx1* and *Isg15*, respectively. Heightened expression of antiviral effectors was specific to *Mx1* and *Isg15*, as *Rnasel* (Ribonuclease L) and *Pkr* (Protein kinase R) expression was comparable in control and knockout animals infected with IAV (Fig 5H).

### Setdb2 expression does not influence transcription factor activation in BM-Mϕ

An augmented antiviral response in *Setdb2* ^-/-^ BM-Mϕ was associated with higher expression of a variety of upstream genes linked to the JAK-STAT, TBK1-IRF7, and Iκκ-NF-κB signaling pathways (Fig 5A). This correlated with upregulation of PRRs, as well as downstream signaling molecules that drive the induction of IFN-I and other proinflammatory cytokines and chemokines. This included upregulation of *Ddx58*, *Ifih1*, *Aim2*, *Nlpr3*, *Tlr3*, *Tlr8*, *Tlr9*, *Mavs*, *Irf7*, *Myd88*, *Stat1* and multiple other genes. Moreover, elevated *Mx1* mRNA in knockout BM-Mϕ was associated with enhanced recruitment of STAT1 and IRF7 to the gene promoter. Thus, we postulated that Setdb2 indirectly suppresses proinflammatory gene expression by inhibiting the activation and nuclear translocation of transcription factors. Despite a 3.4-fold increase in *Stat1* mRNA (Fig 5A), no difference in total STAT1 protein based on absorbance at 450-nm was observed in knockout BM-Mϕ stimulated treated a media alone (2.31 ± 0.12 vs. 2.39 ± 0.09) or IFN-I (2.32 ± 0.07 vs. 2.30 ± 0.11). At 30 minutes post-cytokine treatment, we unable to detect phosphorylated STAT1 in whole-cell extracts in either group. In contrast to STAT1, a higher concentration of NF-κB p65 protein was detected in knockout BM-Mϕ (211.4 ± 17.64 ng/mL vs. 290 ± 16.6 ng/mL; *p*<0.01). However, no correlation between Setdb2 expression and NF-κB p65 activation was observed, as an equal concentration of phosphorylated NF-κB p65 was detected in whole-cell lysates from control and knockout BM-Mϕ treated with a vehicle control (16.92 ± 0.89 ng/mL vs. 18.84 ± 3.21 ng/mL) or stimulated with IFN-I (21.29 ± 1.27 ng/mL vs. 24.65 ± 1.66 ng/mL) for a half hour.

### Setdb2 expression by myeloid cells controls inflammatory cell recruitment in IAV infection

Since several chemokines were upregulated in *Setdb2* ^-/-^ BM-Mϕ, we asked if Setdb2 controls the influx of immune cells to the lungs. *Setdb2* ^*ff*^ *Lyz2* ^*cre+*^ mice had a 2-fold increase in the number of inflammatory monocytes and alveolar Mϕ on day 4 post-infection (Fig 6A). More CCR2^+^ monocytes in knockout lungs correlated with an increase in *Ccl2* transcript and protein, a potent chemotactic mediator of inflammatory monocytes (Fig 6B and 6C). Similar to the lungs, *Setdb2* ^-/-^ BM-Mϕ stimulated with IFN-I transcribed more *Ccl2* than control cells (Fig 6D) and ChIP analysis revealed *Ccl2* is a Setdb2 target gene (Fig 6E and 6F). In respect to control cells, a 10- and 30-fold reduction in Setdb2 and H3K9me3, respectively, was observed in the *Ccl2* promoter in *Setdb2* ^-/-^ BM-Mϕ (Fig 6E and 6F).

### Setdb2 expression by Mϕ influences T cell proliferation in IAV-infected lungs

**Fig 7:**
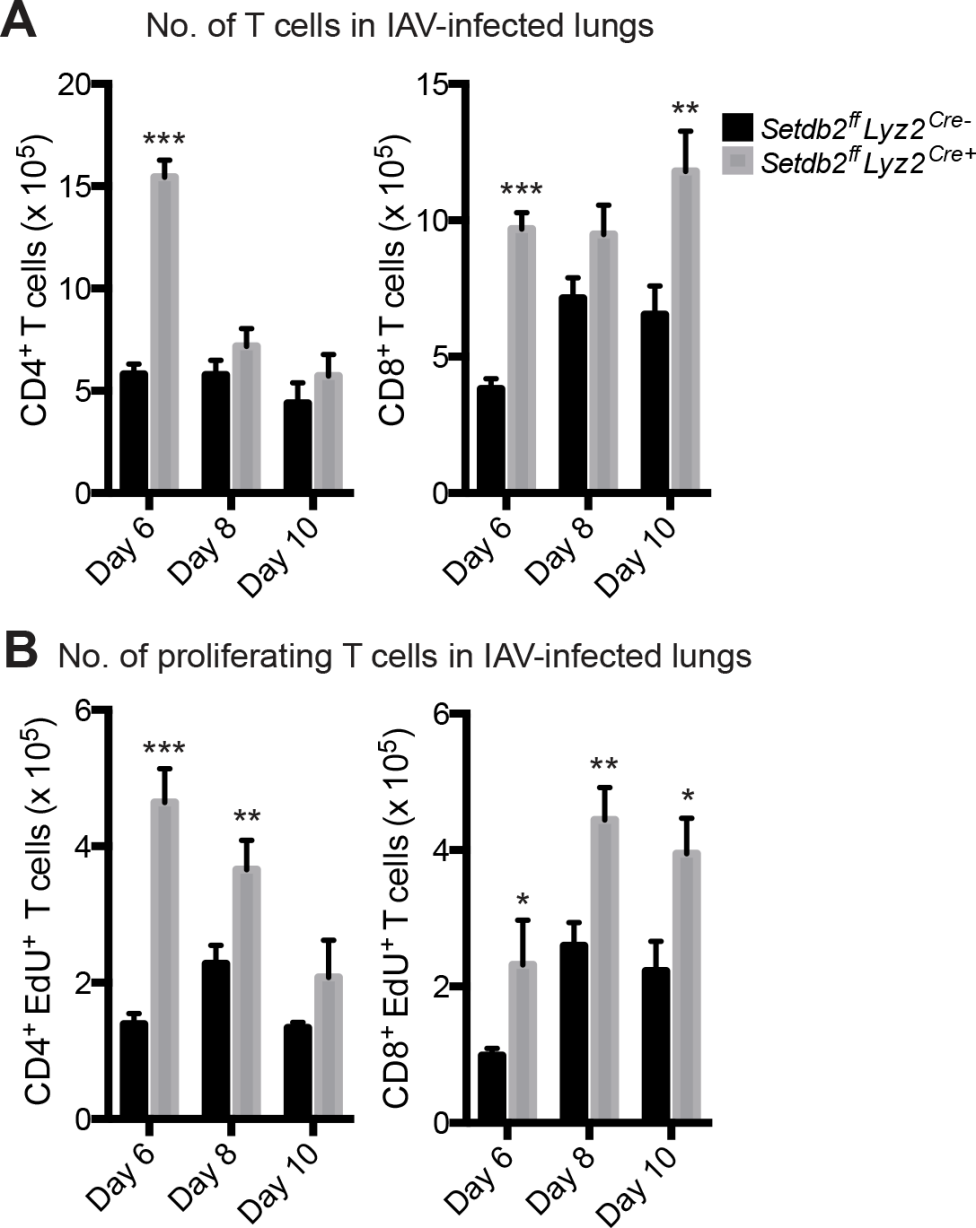
Setdb2 expression by Mϕ influences T cell proliferation in IAV-infected lungs. **(A, B)** Control and *Setdb2* ^*ff*^ *Lyz2* ^*cre+*^ mice infected with 1 x 10^4^ PFU of IAV and euthanized on days 6, 8, and 10 post-infection. **(A)** The number of CD4^+^ and CD8^+^ T cells in IAV-infected lungs. **(B)** To measure proliferation, mice were injected with the thymidine analogue EdU one day prior to euthanization. The number of proliferating (EdU^+^) CD4^+^ and CD8^+^ T cells was calculated over the indicated time course. Values represent mean ± SEM (n = 5–20 mice) from 2–4 independent experiments. *p<0.05; **p<0.01; ***p<0.001 (relative to control lungs).

Transcriptional analysis of IFN-I-stimulated BM-Mϕ identified Setdb2 as a negative regulator of antiviral immunity. This included the suppression of a number of cytokines and chemokines implicated in the migration and subsequent activation of T cells in the lungs following viral infection (Fig 5A and 5B). Although the number of CD4^+^ and CD8^+^ T cells in IAV-infected lungs was comparable on day 4 post-infection (Fig 6A), it is possible that this time point occurred prior to the initiation of the adaptive immune system. This is supported by the observation that reduced disease severity in *Setdb2* ^*ff*^ *Lyz2* ^*Cre+*^ mice was not observed until day 8 post-infection. To address this concern, control and *Setdb2* ^*ff*^ *Lyz2* ^*Cre+*^ mice infected with a sublethal dose of IAV were euthanized on days 6, 8, and 10 post-infection to determine the proportion and number of T cells in the lungs. In both groups, the proportion of CD4^+^ T cells represented 10 to 15% of cells in the lungs at all days examined. A very modest, yet significant increase in CD4^+^ T cells was observed in knockout lungs in comparison to controls (9.73 ± 0.70% vs. 12.90 ± 1.02%; *p*<0.05). In contrast, the proportion of CD8^+^ T cells increased over time in IAV-infected lungs. On day 6 post-infection, the proportion of CD8^+^ T cells were comparable in control and mutant lungs, with CD8^+^ T cells composing approximately 6% of total cells. Relative to wild-type mice, knockout mice had an increase proportion of CD8^+^ T cells in the lungs on day 8 (13.25 ± 1.00% vs. 16.87 ± 0.79%; *p*<0.01) and day 10 (15.92 ± 1.44% vs. 21.32 ± 1.37%; *p*<0.05). Despite only modest changes in the percentage of T cells, the absolute number of T cells was significantly altered in the absence of myeloid *Setdb2*. Notably, *Setdb2* ^*ff*^ *Lyz2* ^*Cre+*^ mice had 3 times the number of both CD4^+^ T cells (5.81 ± 0.50 x 10^5^ vs. 15.43 ± 0.84 x 10^5^; *p*<0.001) and CD8^+^ T cells (3.82 ± 0.37 x 10^5^ vs. 9.69 ± 0.59 x 10^5^; *p*<0.001) in the lungs on day 6 post-infection. While the number of both T cell populations were similar in control and knockout lungs on day 8 post-infection, a 2-fold increase in CD8^+^ T cells (6.54 ± 1.05 x 10^5^ vs. 11.80 ± 1.47 x 10^5^; *p*<0.01) was observed on day 10 in mice lacking *Setdb2* (Fig 7A).

Following homing to the lungs, IAV triggers the activation and subsequent expansion of T cells at the site of infection. Since knockout mice had a greater number of both CD4^+^ and CD8^+^ T cells, we asked whether changes in proliferative capability contributed to the altered T cell prolife in IAV-infected lungs. To determine the extent of expansion, IAV-infected mice were injected with EdU (5-ethynyl-2'-deoxyuridine) and euthanized on the indicated days. While proliferation of both CD4^+^ and CD8^+^ T cells peaked on day 8 post-infection, the extent of EdU incorporation was significantly higher in knockout lungs. In wild-type lungs, 39.55 ± 2.06% of CD4^+^ T cells and 34.72 ± 2.20% of CD8^+^ T cells were EdU positive. In the absence of myeloid *Setdb2*, EdU incorporation was enhanced by greater than 10% in both CD4^+^ T cells (39.55 ± 2.06% vs. 51.00 ± 2.62%; *p*<0.01) and CD8^+^ T cells (34.72 ± 2.20% vs. 48.20 ± 1.83%; *p*<0.01). In contrast, gating on either CD4^+^ or CD8^+^ T cells on days 6 and 10 post-infection revealed no changes in the extent of EdU incorporation between the two groups of animals. However, despite this observation, the absolute number of proliferating T cells was enhanced on two or all three time points depending on the T cell subset. In respect to controls, knockout lungs had approximately 3 x 10^5^ and 1 x 10^5^ more CD4^+^ EdU^+^ T cells in the lungs on days 6 and 8 post-infection, respectively. Similarly, *Setdb2* ^*ff*^ *Lyz2* ^*Cre+*^ mice had a greater number of CD8^+^ EdU^+^ T cells in the lungs at each time point examined. The greatest difference in cell number was observed on day 8 post-infection (2.60 ± 0.34 x 10^5^ vs. 4.44 ± 0.47 x 10^5^; *p*<0.01), with nearly 2 x 10^5^ more proliferating CD8^+^ T cells in IAV-infected lungs. A significant increase in the number of CD8^+^ EdU^+^ T cells was also observed in knockout lungs on day 6 (0.99 ± 0.11 x 10^5^ vs. 2.32 ± 0.66 x 10^5^; *p*<0.05) and day 10 (2.22 ± 0.44 x 10^5^ vs. 3.95 ± 0.52 x 10^5^; *p*<0.05) post-infection (Fig 7B).

### Setdb2 expression by Mϕ influences antigen-specific cytokine production by CD4^+^ T cells

Mϕ, along with DCs and B cells, process and subsequently present viral antigen to CD4^+^ T cells to stimulate the production of cytokines with antiviral and/or immunomodulatory activity. This prompted us to ask whether *Setdb2* expression by Mϕ influences antigen presentation and downstream T cell responses. Since this process is dependent on the expression of MHC class II, as well as several co-stimulatory molecules, we initially examined cell surface markers on IFN-I-stimulated BM-Mϕ by flow cytometry. The level of MHC class II, CD40, and CD86 expression on the cell surface was comparable in both groups. In contrast, changes in IFNAR and CD80 expression were observed in the absence of *Setdb2*. Based on MFI, IFNAR was reduced (959.0 ± 7.0 vs. 679.5 ± 10.5; *p<*0.001) and CD80 was elevated (1868.0 ± 8.0 vs. 2464.8 ± 58.4; *p<*0.001) in *Setdb2* ^-/-^ BM-Mϕ stimulated with IFN-I. Despite similar levels on the cell surface, greater than a 2-fold increase in *Cd40* and *Cd86* transcript was detected in *Setdb2* ^-/-^ BM-Mϕ stimulated with IFN-I (Fig 5A).

**Fig 8:**
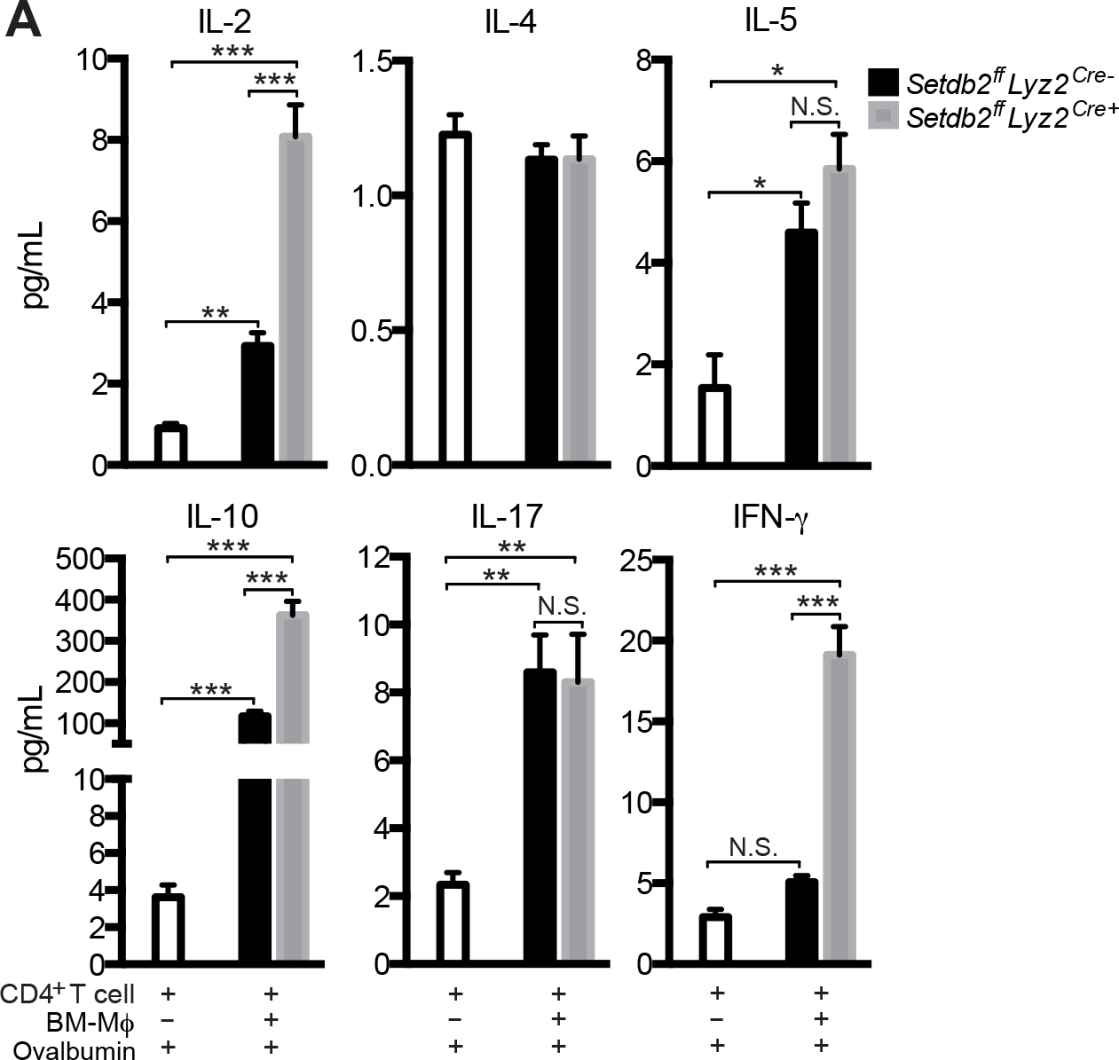
A. Setdb2 expression by Mϕ influences antigen-specific cytokine production by CD4^+^ T cells. **(A)** CD4^+^ T cells isolated from OT-II mice were cultured with ovalbumin peptide in the absence or presence of control or *Setdb2* ^-/-^ BM-Mϕ. The concentration of IL-2, IL-4, IL-5, IL-10, IL-17, and IFN-γ in supernatants was measured 48 hours after plating. Values represent mean ± SEM from 3–4 independent experiments. *p<0.05; **p<0.01; ***p<0.001.

To examine antigen-specific T cell responses, CD4^+^ T cells from naive OT-II mice were cultured alone or at a 5:1 ratio with either wild-type or *Setdb2* ^-/-^ BM-Mϕ stimulated with IFN-I in the presence of ovalbumin peptide. When co-cultured with either control or knockout BM-Mϕ, CD4^+^ T cells produced more IL-2, IL-5, IL-10, and IL-17 (Fig 8A). While the concentration of IL-5 and IL-17 was comparable between groups, the absence of *Setdb2* in BM-Mϕ was associated with enhanced IL-2 and IL-10 production (Fig 8A). In contrast, IFN-γ production was only enhanced in co-cultures containing *Setdb2* ^-/-^ BM-Mϕ (Fig 8A). This correlated with a 4-fold increase in IFN-γ relative to CD4^+^ T cells cultured alone or with control BM-Mϕ. IL-4 was the only cytokine examined that was unaltered in all experimental conditions (Fig 8A).

## Discussion

This study uncovered a novel role for a histone-modifying enzyme in regulating immunity in acute respiratory viral infection. We have demonstrated IFN-I-dependent induction of *Setdb2* in myeloid cell, most notably alveolar Mϕ and inflammatory monocytes, controls the severity of IAV infection by suppressing innate and adaptive immune responses.

Robust production of IFN-I is required to control infection with several viral pathogens. IFN-I signaling results in the transcription of ISGs involved in antiviral immunity. We identified IFN-I as a potent inducer of *Setdb2* expression in Mϕ. The induction of *Setdb2* by IFN-I was dependent on the JAK-STAT pathway, as blocking JAK1 or STAT1 activity diminished expression. IFN-γ and IFN-λ also signal through STAT1; however, these cytokines had little to no effect on *Setdb2* expression, suggesting a downstream molecule in the signaling cascade drives IFN-I-dependent expression. In addition to STAT1, the transcription factor IRF7 regulated *Setdb2* expression in in BM-Mϕ. While both STAT1 and IRF7 bound to the *Setdb2* promoter, it is unclear if the transcription factors independently regulate transcription or work in concert for optimal expression. It is somewhat unexpected that IFN-λ did not upregulate *Setdb2* since IFN-I and IFN-III have overlapping roles in viral infection and despite signaling through different receptors, initiate a similar signaling cascade resulting in ISGF3 activation [34]. The degree of *Setdb2* transcription correlated with the concentration of IFN-I, suggesting the extent of expression may be a useful biomarker for determining the severity of infection and autoimmune diseases that result in copious IFN-I production.

Alveolar Mϕ are the first line of defense against inhaled pathogens and are required to control IAV infection [2–5]. We found that the majority of alveolar Mϕ in IAV-infected lungs expressed Setdb2 and mice deficient for Setdb2 in Mϕ displayed prolonged survival when challenged with a lethal dose of IAV. One mechanism by which alveolar Mϕ control infection is through the production of IFN-I, which, in turn, induces the expression of antiviral effectors that inhibit viral replication and dissemination. It is well documented that IFN-I-driven induction of Mx1, a dynamin-like GTPase that blocks viral transcription and replication, is critical for controlling IAV infection [35]. In addition to Mx1, the ubiquitin-like protein ISG15 has been shown to have an important role in protecting cells from viral pathogens. In IAV infection, ISG15 limits viral replication by binding to the N-terminal RNA-binding domain of NS1, blocking nuclear import [36]. Setdb2 selectively repressed the expression of *Mx1* and *Isg15* in BM-Mϕ stimulated with IFN-I and accelerated viral clearance in *Setdb2* ^*ff*^ *Lyz2* ^*cre+*^ lungs correlated with enhanced transcription of both genes. Thus, by regulating the expression of specific ISGs, Setdb2 dictated the severity of infection. Since the selectivity of the identified Setdb2-regulated antiviral genes is not limited to IAV, it is plausible that Setdb2 controls the resolution of infection caused by other viral pathogens.

IFN-I upregulated *Setdb2* in dose-dependent manner and, in turn, Setdb2 regulated the amplitude of the IFN-I response. We propose three mechanisms by which Setdb2 may repress the expression of IFN-I and downstream ISGs. First, Setdb2 regulated the expression of key transcription factors in the IFN-I signaling cascade, including *Stat1* and *Irf7*. Second, Setdb2 may control the antiviral response by regulating the expression of PRRs since viral sensing by the innate immune system results in cytokine and chemokine production. Enhanced expression of the RNA helicases *Ddx58* and *Ifih1*, as well as downstream genes including *Tbk1*, *Mavs*, *Myd88*, *Irf7*, among others was observed in *Setdb2* ^-/-^ BM-Mϕ This finding suggests Setdb2 suppresses these genes to weaken the IFN-I response. Third, in addition to targeting viral proteins, ISG15 can bind to IFN-associated transcription factors and antiviral effectors to enhance innate immunity [37–39], suggesting Setdb2-dependent regulation of ISG15 may reduce the stability of components involved in IFN-I production and downstream responses. Although IFN-I is essential for controlling IAV infection, its production must be tightly regulated to prevent tissue damage. Multiple genes involved in the suppression of IFN-I were upregulated in parallel with *Ifna2* and *Ifnb1* in *Setdb2* ^-/-^ BM-Mϕ. One example is *Pin1* (Peptidyl-prolyl cis-trans isomerase NIMA-interacting 1), which negatively regulates the antiviral response by promoting the degradation of IRF3 [40]. The transcription factor Foxo3 (Forkhead box O3) negatively regulates the expression of antiviral genes by forming a regulatory circuit involving IRF7 and IFN-I [41]. The expression profile of antiviral genes in *Setdb2* ^-/-^ BM-Mϕ closely mimicked *Foxo3* ^-/-^ BM-Mϕ. This includes enhanced expression of *Ccl5*, *Irf7*, *Ifnb1*, *Ddx58*, *Stat1*, as well as additional ISGs suggesting Setdb2 may have a similar role in balancing the beneficial and detrimental consequences of IFN-I.

In addition to IFN-I, Setdb2 influenced the expression of proinflammatory cytokines and the immunoregulatory cytokine IL-10. Of the cytokines examined, TNF-α was the only one repressed in *Setdb2* ^-/-^ BM-Mϕ. This finding is of interest based on reports demonstrating cross-regulation of IFN-I and TNF-α. It is unclear whether Setdb2 promotes TNF-α expression directly or indirectly through other proteins that regulate transcription and/or translation. Since tri-methylation of H3K9 imprints a repressive mark in chromatin, it is unlikely that Setdb2 directly induces *Tnf* transcription. Indirect regulation of TNF-α may be cytokine-dependent, as reduced TNF-α was associated with overexpression of IFN-I and IL-10. While it is unclear if IFN-I itself can directly suppress TNF-α expression, IL-10 dampens expression by inhibiting the activation of NF-κB [42]. Another possibility is that Setdb2 influences TNF-α expression by regulating proteins that repress transcription. For example, Twist proteins bind to the *Tnf* promoter to inhibit transcription and IFN-I suppresses expression by activating the receptor tyrosine kinase Axl upstream of Twist1/2 [43]. Finally, IFNs can suppress TNF-α expression in Mϕ by promoting tristetraprolin-mediated mRNA decay in a STAT1- and p38-dependent manner, suggesting Setdb2 may negatively regulate the expression of tristetraprolin or similar proteins [44]. Although IAV infection results in robust TNF-α production, it is unnecessary for viral clearance. Rather, TNF-α, along with Nos2, is the major culprit of immunopathology [45,46]. Furthermore, imbalanced production of either IFN-I or TNF-α is linked to autoimmunity. Rheumatoid arthritis patients, as well as children with chronic arthritis being treated with TNF-α antagonists can develop lupus-like symptoms due to overexpression of IFN-α and ISGs [47,48]. Therefore, targeting Setdb2 in parallel with anti-TNF-α therapy may be beneficial for repressing exaggerated IFN-I activity in autoimmunity.

Mortality in individuals exposed to highly pathogenic strains of IAV is often due to lung injury, rather than uncontrolled viral replication. Infection caused by respiratory pathogens that target epithelial cells results in significant cell death and as a consequence, the airways become clogged with dead cells, cellular debris, surfactant material, and virus. Respiratory failure characterized by defective gas exchange and fatal hypoxia is observed in mice lacking alveolar Mϕ [21], highlighting the importance of sustained Mϕ viability in infection. Consistent with reports demonstrating that the level of morbidity in IAV infection is dependent on the number of resident Mϕ [49,50], a greater number of alveolar Mϕ in *Setdb2* ^*ff*^ *Lyz2* ^*cre+*^ mice correlated with less damage to the airways and prolonged survival. There are several potential mechanisms by which Setdb2 may dictate the number of alveolar Mϕ. First, fewer alveolar Mϕ in control mice may indicate Setdb2 regulates genes that promote cell death. In IAV infection, the majority of resident Mϕ are depleted by one week due to necrosis [51], indicating Setdb2 expression by alveolar Mϕ may facilitate the necrotic process. Elevated *Ccl5* expression may promote Mϕ survival since the CCL5-CCR5 nexus inhibits apoptosis. Accordingly, mortality is increased in *Ccl5* ^-/-^ or *Ccr5* ^-/-^ in IAV infection [19]. Second, Setdb2 may inhibit Mϕ proliferation by suppressing local GM-CSF production [52]. Third, since alveolar Mϕ can arise from blood monocytes [53], enhanced infiltration in the absence of *Setdb2* may re-populate the number of alveolar Mϕ in IAV infection. Furthermore, since the removal of apoptotic cells, cellular debris, and surfactant material limits tissue damage, Setdb2 may control the extent of injury by diminishing the phagocytic capacity of Mϕ. Collectively, Setdb2 expression by Mϕ may control IAV-induced lethality by regulating airway integrity and minimizing tissue damage.

Alveolar Mϕ are a major source of chemokines following infection and as a result, promote the infiltration of inflammatory cells to the lungs. The CCR2-CCL2 axis regulates the emigration of monocytes from the bone marrow and subsequent recruitment to infectious sites [54]. An enhanced number of CCR2^+^ monocytes correlated with more CCL2 in *Setdb2* ^*ff*^ *Lyz2* ^*Cre+*^ lungs and Setdb2 directly regulated *Ccl2* transcription in BM-Mϕ However, despite this clear correlation, it does not exclude the possibility that *Setdb2* ^-/-^ cells are highly potent in chemotaxis. In tissue, monocytes differentiate into DCs and exudate Mϕ and these cells are linked to severe disease due to overwhelming proinflammatory cytokine production [45,46,55,56]. In contrast, we found that enhanced monocyte recruitment was associated with prolonged survival. How knockout mice avoided immunopathology needs to be further explored. Although the aforementioned studies implicate monocyte-derived cells as the culprit of IAV-induced tissue damage and death, others have shown that tissue Mϕ can restore lung homeostasis and limit injury by developing an immunoregulatory phenotype that resembles that of alveolar Mϕ [57]. In addition to monocytes, neutrophils cause significant tissue damage in IAV infection. It has been proposed that IFN-I-dependent generation of monocytes attenuates neutrophil infiltration and as a consequence, reduces tissue damage [58]. Consistent with a recent study, the absence of *Setdb2* in BM-Mϕ resulted in increased expression of neutrophil chemoattractants [59]. However, neutrophil recruitment was comparable in control and *Setdb2* ^*ff*^ *Lyz2* ^*Cre+*^ lungs. This inconsistency may be due to the model system, as neutrophils are more critical in bacterial infection. Moreover, the mouse strain used may account for the discrepancy. Whereas they used mice deficient for Setdb2 in all cells, our mice specifically lacked Setdb2 in monocytes, Mϕ, and granulocytes.

In addition to their central role in innate immune responses, Mϕ are involved in the initiation and maintenance of the adaptive immunity. Resident and recruited Mϕ populations can present antigen to CD4^+^ T cells and we showed that Setdb2 expression by Mϕ suppresses T cell proliferation and cytokine production. In IAV infection, Th1 cells are characterized by co-production of IFN-γ and IL-10 [60,61] and CD4^+^ T cells cultured with *Setdb2* ^-/-^ BM-Mϕ secreted more of both cytokines. In addition to IFN-γ and IL-10, CD4^+^ T cells produced more IL-2, which likely contributes to enhanced T cell proliferation in infected lungs. Whether Setdb2 expression regulates CD4^+^ T cell responses through antigen presentation is unclear. Several lines of evidence suggest that Setdb2 may regulate the magnitude of T cell responses indirectly through altered cytokine and chemokine production. The polarization of naïve CD4^+^ T cells to Th1 cells is dependent on IL-12 and *Setdb2* ^-/-^ BM-Mϕ secreted more IL-12p40 than control cells. Moreover, IFN-I and CCL5 can upregulate the expression of co-stimulatory molecules on antigen presenting cells, as well as regulate the phenotype of CD4^+^ T cells by promoting cytokine production and proliferation [62–66]. Since transcription of both genes was enhanced in the absence of Setdb2, heightened IFN-I and CCL5 production may further amplify CD4^+^ T cell responses. Other lymphoid populations, including CD8^+^ T cells and B cells, are important for the eradication of virus. Respiratory pathogens can trigger the formation of densely packed clusters of lymphocytes known as inducible bronchus associated lymphoid tissue (BALT). In IAV infection, BALT primes virus-specific T and B cells in the lungs and as a result, accelerates viral clearance [67]. Lung histology revealed the presence of potential lymphoid structures in infected *Setdb2* ^*ff*^ *Lyz2* ^*cre+*^ mice, but not control animals. Future studies are required to determine if the accumulation of lymphoid cells in knockout lungs is in fact BALT and if it facilitates prolonged survival.

Understanding why a host would upregulate a histone-modifying enzyme that suppresses antiviral immunity is a challenging question. One possible reason is IAV hijacks host machinery to ‘turn on’ *Setdb2*, thereby diminishing IFN-I expression and downstream responses to evade the immune system. By mimicking a sequence in the tail of histone H3, the viral protein NS1 allows H3N2 to utilize transcriptional regulators to suppress the antiviral response [68]. Additionally, NS1 allows IAV to evade the immune system by preventing apoptosis through PI3K (phosphatidylinositide 3-kinase) activation and limiting IFN-β production by inhibiting IRF3 activity [69,70]. Recently, it was demonstrated that IAV escapes the IFN-I response by triggering the production of prostaglandin E_2_ by Mϕ, which suppresses both innate and adaptive immunity allowing the virus to replicate more efficiently [71]. It is also plausible that even if Setdb2 suppresses antiviral immunity, it is not dramatic enough to cause significant damage to the host. IAV-associated death is rarely caused by primary infection alone. Rather, secondary bacterial pneumonia is the leading cause of mortality caused by an infectious agent [72]. While the mechanism responsible for enhanced susceptibility to bacterial superinfection is not fully elucidated, IFN-I sensitizes the host to bacterial pneumonia [73–75]. Since Setdb2 regulates the IFN-I response in viral infection, it is possible the host has evolved to diminish the antiviral response enough to control viral infection, yet prevent secondary complications. While preparing this manuscript, it was shown that Setdb2 regulates the crosstalk between IFN-I and the NF-κB pathway to control the neutrophil response in bacterial superinfection [59]. Together with our findings, these results implicate Setdb2 as a promising therapeutic target in respiratory viral infection and potentially, in secondary complications and autoimmune diseases linked to IFN-I activity.

## Materials and Methods

### Ethics statement

All animal procedures were approved by the University Committee on the Use and Care of Animals at the University of Michigan (PRO00004191) and done in accordance with the Animal Welfare Act guidelines of the National Institutes of Health. Experiments using human samples were approved by the Institutional Review Board of the University of Michigan (HUM00075841) and conducted in accordance with the principles expressed in the Declaration of Helsinki. Written informed consent was obtained from all adult subjects.

### Mice and IAV infection

C57BL/6, 129S5, and *Stat1* ^-/-^ mice were purchased from Taconic (Germantown, NY). B6.129P2-*Lyz2* ^*tm1(cre)Ifo*^/J (*Lyz2* ^*cre*^ mice), and B6.Cg-Tg(TcraTcrb)425Cbn/J (OT-II) transgenic mice were purchased from The Jackson Laboratory (Bar Harbor, ME). *Setdb2* gene targeted embryonic stem (ES) cell clones EPD0164_4-B10, -E09, and -E12 were obtained from the trans-NIH Knockout Mouse Project (KOMP Repository). The JM8.N4 C57BL/6N ES cell clones [76] carried the knockout first tm1a(KOMP)Wtsi *Setdb2* allele [77]. The ES cells were expanded in cell culture and chromosome counts were performed. Correct targeting of the *Setdb2* gene was confirmed by genetic analysis of DNA from the ES cell clones. Germline transmission of the *Setdb2* ^*tm1a*^ allele was obtained by breeding ES cell-mouse chimeras produced by the microinjection of C57BL/6/BrdCrHsd-*Tyr* ^*c*^ albino C57BL/6 blastocysts with the ES cell clones. Chimeras were mated with FLPo recombinase mice to remove the drug selection cassette and produce mice carrying the conditional floxed *Setdb2* ^*tm1c*^ allele. C57BL/6-*Tg(CAG-Flpo)* ^*1Afst*^/Mmucd FLPo recombinase mice [78] were obtained from the Mutant Mouse Resource and Research Centers (Stock Number: 032247-UCD). FLPo mice were backcrossed onto albino C57BL/6 mice so that coat color selection could be used to chimeras to identify germline transmission and maintain an inbred C57BL/6 genetic background. The resulting progeny with a floxed *Setdb2* allele were bred with *Lyz2* ^*cre*^ mice to generate control and mice deficient for *Setdb2* in monocytes, Mϕ, and granulocytes [79]. *Setdb2* ^*ff*^ *Lyz2* ^*cre*^ and *Setdb2* ^*LacZ*^ reporter mice were bred in-house and genotyped with custom primers (S1 Table). For infection, the IAV strain A/PR8/34; H1N1 isotype was used (ATCC). Age-matched female mice were inoculated intranasally with 1 x 10^4^ PFU for sublethal infection and 1 x 10^5^ PFU for lethal infection.

### Cell isolation

Murine CD11b microbeads, human CD14 microbeads, and the murine CD4^+^ T cell Isolation Kit were purchased from Miltenyi Biotec. Magnetic separation yielded 95% purity of each population. PBMCs were isolated from the blood using Ficoll (GE Healthcare).

### BM-Mϕ and human Mϕ differentiation

For the generation of BM-Mϕ, bone marrow was differentiated in L929 cell-conditioned media as previously described [80]. For the differentiation of human of Mϕ, CD14^+^ monocytes were cultured in complete medium supplemented with 50 ng/mL of M-CSF (R&D systems) for one week. Adherent cells were washed and harvested with Trypsin/EDTA (Lonza).

### Epithelial cell culture

Murine AECs were isolated from naïve mice as previously described [81]. Briefly, Dispase-digested lungs were depleted of CD16/CD32^+^ and CD45^+^ cells using biotinylated antibodies (BD Biosciences) and anti-biotin microbeads (Miltenyi Biotec). Non-adherent cells were cultured in fibronectin-coated wells and AECs were harvested after 4 days. NHBEs and the BEGM BulletKit were purchased from Lonza. NHBEs were cultured in 25 cm^2^ flasks following the manufacturers recommendations. NHBEs were subcultured using Trypsin/EDTA when 80% confluent.

### Cytokine and tofacitinib treatment

Murine IFN-α, IFN-β, IFN-γ, IFN-λ2, and IFN-λ3, as well as human IFN-α and IFN-β were purchased from R&D Systems. For IFN-I treatment, cells were given 10 units/mL of IFN-α and IFN-β unless otherwise noted. Cells were treated with 10 ng/mL of IFN-II (IFN-γ) or IFN-III (IFN-λ2 and IFN-λ3). For JAK inhibition, cells were treated with 50 nM tofacitinib (Cayman Chemical) at the time of stimulation.

### siRNA

IRF3 and IRF7 siRNA were purchased from Santa Cruz Biotechnology. Non-targeting siRNA was purchased from GE Dharmacon. BM-Mϕ were transfected with siRNA using the Amaxa Mouse Mϕ Nucleofactor Kit (Lonza). Transfected cells were cultured for 18 hours before stimulation.

### Mϕ-T cell co-culture

CD4^+^ T cells isolated from the spleens of naïve OT-II mice were cultured alone or with BM-Mϕ at a 5:1 ratio. For cytokine analysis, co-cultures were incubated for 48 hours in the presence of 10 ng/mL of ovalbumin peptide (Peptides International).

### RT-PCR

RNA was extracted using TRIzol Reagent (Invitrogen) and cDNA was generated with the iScript cDNA synthesis kit (Bio-Rad). TaqMan primer/probe sets for murine *Setdb2*, *Ifna2*, *Ifnb1*, *Irf3*, *Irf7*, *Ccl2*, *Cxcl1*, *Cxcl2*, *Il10*, *Tnf*, *Il10*, *Tnf*, *Pkr*, *Rnasel*, *Isg15*, *Mx1*, and human *SETDB2* were purchased from Applied Biosystems. *NS1* and *M1* were detected using custom primers [5]. Gene expression was assessed using an ABI Prism 7500 instrument (Applied Biosystems) and normalized to *Gapdh* or *ACTB*.

### PCR arrays

The murine/human epigenetic chromatin-modifying enzyme and murine antiviral PCR arrays were purchased from SABiosciences. RNA was DNase-digested using the RNeasy Mini Kit and reverse transcribed with the RT^2^ First Strand Kit (Qiagen). RT-PCR was performed according to the manufacturer’s instructions and gene expression was normalized to multiple housekeeping genes.

### Cytokine and chemokine production

The concentration of CCL2 was measured using the mouse CCL2 ELISA Ready-SET-Go! (eBioscience). All other cytokines were quantified using a Bio-Plex 200 (Bio-Rad Laboratories).

### ChIP

A total of 1 x 10^7^ BM-Mϕ were treated with a vehicle control or cytokine for 24 hours and ChIP was performed as previously described [24]. DNA was fragmented by sonication using a Branson Sonifier 450 (Branson Ultrasonics). For immunoprecipitation, the following antibodies were used: IRF7 (Santa Cruz Biotechnology), STAT1 (Abcam), H3K9me3 (Abcam), Setdb2 (Dr. Yali Dou, University of Michigan), and rabbit polyclonal IgG (Millipore). DNA was assessed by RT-PCR using custom primers (S2 Table).

### STAT1 and NF-κB expression

For transcription factor analysis, whole-cell lysates were collected from a total of 1 x 10^7^ BM-Mϕ treated with a vehicle control or IFN-I for 30 minutes. The concentration of protein in each sample was adjusted to a concentration of 100 μg/mL in assay buffer. Total and phosphorylated protein was measured using the following kits from abcam: STAT1 (pY701) + total STAT1 ELISA Kit, STAT1 (pS727) + total STAT1 ELISA Kit, and NF-κB p65 (pS536) + total NF-κB p65 SimpleStep ELISA Kit.

### Flow cytometry

Lungs were digested in RPMI 1640-based complete medium containing 1 mg/mL of collagenase (Roche) and 30 μg/mL DNase I (Sigma-Aldrich). Samples were passed through an 18-gauge needle, filtered, stained with the LIVE/DEAD Fixable Violet Dead Cell Stain Kit (Life Technologies), blocked with anti-CD16/32, and stained with the indicated antibodies. Antibodies were purchased from eBioscience (CD11b, CD11c, F4/80, Ly6C, Ly6G, CD3, CD8, NK1.1, and IFNAR1), R&D systems (CCR2), and BD Biosciences (MHC-II, CD80, CD86, and CD40). To characterize *lacZ* expression, the *Fluo*Reporter LacZ Flow Cytometry Kit was used (Life Technologies). For T cell proliferation, mice were intraperitoneally given 10 mg/mL EdU one day prior to euthanization. Following surface staining, cells were labeled according to the manufacturer’s protocol. Data were acquired on a LSR II (BD Biosciences) and analyzed with FlowJo software (TreeStar).

### Histology

Lungs were inflated with 10% formalin and processed using routine histological techniques. Tissue sections were stained with H&E and visualized by light microscopy.

### Statistical analysis

For the survival study, the *p* value was determined by a log-rank survival test. Analysis of the antiviral gene profile in BM-Mϕ was characterized by one-way ANOVA. Differences for remaining experiments were analyzed by Student’s *t*-test or two-way ANOVA. A *p* value of ≤0.05 was considered significant.

## Supporting Information

S1 Table Genotyping primers and PCR conditions. (PDF)

S2 Table Custom ChIP primers. (PDF)

## References

[1] Palese P (2004) Influenza: old and new threats. Nat Med 10: S82–87. 15577936 10.1038/nm1141

[2] Tumpey TM, Garcia-Sastre A, Taubenberger JK, Palese P, Swayne DE, et al (2005) Pathogenicity of influenza viruses with genes from the 1918 pandemic virus: functional roles of alveolar macrophages and neutrophils in limiting virus replication and mortality in mice. J Virol 79: 14933–14944. 16282492 10.1128/JVI.79.23.14933-14944.2005 PMC1287592

[3] Tate MD, Pickett DL, van Rooijen N, Brooks AG, Reading PC (2010) Critical role of airway macrophages in modulating disease severity during influenza virus infection of mice. J Virol 84: 7569–7580. 10.1128/JVI.00291-10 20504924 PMC2897615

[4] Kim HM, Lee YW, Lee KJ, Kim HS, Cho SW, et al (2008) Alveolar macrophages are indispensable for controlling influenza viruses in lungs of pigs. J Virol 82: 4265–4274. 10.1128/JVI.02602-07 18287245 PMC2293066

[5] Ito T, Allen RM, Carson WFt, Schaller M, Cavassani KA, et al (2011) The critical role of Notch ligand Delta-like 1 in the pathogenesis of influenza A virus (H1N1) infection. PLoS Pathog 7: e1002341 10.1371/journal.ppat.1002341 22072963 PMC3207886

[6] Kumagai Y, Takeuchi O, Kato H, Kumar H, Matsui K, et al (2007) Alveolar macrophages are the primary interferon-alpha producer in pulmonary infection with RNA viruses. Immunity 27: 240–252. 17723216 10.1016/j.immuni.2007.07.013

[7] Becker S, Quay J, Soukup J (1991) Cytokine (tumor necrosis factor, IL-6, and IL-8) production by respiratory syncytial virus-infected human alveolar macrophages. J Immunol 147: 4307–4312. 1753101

[8] Takeuchi O, Akira S (2010) Pattern recognition receptors and inflammation. Cell 140: 805–820. 10.1016/j.cell.2010.01.022 20303872

[9] Honda K, Yanai H, Negishi H, Asagiri M, Sato M, et al (2005) IRF-7 is the master regulator of type-I interferon-dependent immune responses. Nature 434: 772–777. 15800576 10.1038/nature03464

[10] Der SD, Zhou A, Williams BR, Silverman RH (1998) Identification of genes differentially regulated by interferon alpha, beta, or gamma using oligonucleotide arrays. Proc Natl Acad Sci U S A 95: 15623–15628. 9861020 10.1073/pnas.95.26.15623 PMC28094

[11] Doherty PC, Turner SJ, Webby RG, Thomas PG (2006) Influenza and the challenge for immunology. Nat Immunol 7: 449–455. 16622432 10.1038/ni1343

[12] Peiris JS, Yu WC, Leung CW, Cheung CY, Ng WF, et al (2004) Re-emergence of fatal human influenza A subtype H5N1 disease. Lancet 363: 617–619. 14987888 10.1016/S0140-6736(04)15595-5 PMC7112424

[13] Beigel JH, Farrar J, Han AM, Hayden FG, Hyer R, et al (2005) Avian influenza A (H5N1) infection in humans. N Engl J Med 353: 1374–1385. 16192482 10.1056/NEJMra052211

[14] Pribul PK, Harker J, Wang B, Wang H, Tregoning JS, et al (2008) Alveolar macrophages are a major determinant of early responses to viral lung infection but do not influence subsequent disease development. J Virol 82: 4441–4448. 10.1128/JVI.02541-07 18287232 PMC2293049

[15] Kirby AC, Coles MC, Kaye PM (2009) Alveolar macrophages transport pathogens to lung draining lymph nodes. J Immunol 183: 1983–1989. 10.4049/jimmunol.0901089 19620319 PMC3609601

[16] Macdonald DC, Singh H, Whelan MA, Escors D, Arce F, et al (2014) Harnessing alveolar macrophages for sustained mucosal T-cell recall confers long-term protection to mice against lethal influenza challenge without clinical disease. Mucosal Immunol 7: 89–100. 10.1038/mi.2013.27 23715172

[17] Albert ML, Sauter B, Bhardwaj N (1998) Dendritic cells acquire antigen from apoptotic cells and induce class I-restricted CTLs. Nature 392: 86–89. 9510252 10.1038/32183

[18] Winau F, Weber S, Sad S, de Diego J, Hoops SL, et al (2006) Apoptotic vesicles crossprime CD8 T cells and protect against tuberculosis. Immunity 24: 105–117. 16413927 10.1016/j.immuni.2005.12.001

[19] Tyner JW, Uchida O, Kajiwara N, Kim EY, Patel AC, et al (2005) CCL5-CCR5 interaction provides antiapoptotic signals for macrophage survival during viral infection. Nat Med 11: 1180–1187. 16208318 10.1038/nm1303 PMC6322907

[20] Mok CK, Lee DC, Cheung CY, Peiris M, Lau AS (2007) Differential onset of apoptosis in influenza A virus H5N1- and H1N1-infected human blood macrophages. J Gen Virol 88: 1275–1280. 17374772 10.1099/vir.0.82423-0

[21] Schneider C, Nobs SP, Heer AK, Kurrer M, Klinke G, et al (2014) Alveolar macrophages are essential for protection from respiratory failure and associated morbidity following influenza virus infection. PLoS Pathog 10: e1004053 10.1371/journal.ppat.1004053 24699679 PMC3974877

[22] Gallagher KA, Joshi A, Carson WF, Schaller M, Allen R, et al (2014) Epigenetic Changes in Bone Marrow Progenitor Cells Influence the Inflammatory Phenotype and Alter Wound Healing in Type 2 Diabetes. Diabetes. 10.2337/db14-0872 PMC4375075 25368099

[23] Ishii M, Wen H, Corsa CA, Liu T, Coelho AL, et al (2009) Epigenetic regulation of the alternatively activated macrophage phenotype. Blood 114: 3244–3254. 10.1182/blood-2009-04-217620 19567879 PMC2759649

[24] Kittan NA, Allen RM, Dhaliwal A, Cavassani KA, Schaller M, et al (2013) Cytokine induced phenotypic and epigenetic signatures are key to establishing specific macrophage phenotypes. PLoS One 8: e78045 10.1371/journal.pone.0078045 24205083 PMC3804553

[25] Kruidenier L, Chung CW, Cheng Z, Liddle J, Che K, et al (2012) A selective jumonji H3K27 demethylase inhibitor modulates the proinflammatory macrophage response. Nature 488: 404–408. 10.1038/nature11262 22842901 PMC4691848

[26] Dillon SC, Zhang X, Trievel RC, Cheng X (2005) The SET-domain protein superfamily: protein lysine methyltransferases. Genome Biol 6: 227 16086857 10.1186/gb-2005-6-8-227 PMC1273623

[27] Mabuchi H, Fujii H, Calin G, Alder H, Negrini M, et al (2001) Cloning and characterization of CLLD6, CLLD7, and CLLD8, novel candidate genes for leukemogenesis at chromosome 13q14, a region commonly deleted in B-cell chronic lymphocytic leukemia. Cancer Res 61: 2870–2877. 11306461

[28] Skarnes WC, Auerbach BA, Joyner AL (1992) A gene trap approach in mouse embryonic stem cells: the lacZ reported is activated by splicing, reflects endogenous gene expression, and is mutagenic in mice. Genes Dev 6: 903–918. 1592261 10.1101/gad.6.6.903

[29] Garber K (2011) Pfizer's JAK inhibitor sails through phase 3 in rheumatoid arthritis. Nat Biotechnol 29: 467–468. 10.1038/nbt0611-467 21654650

[30] Honda K, Takaoka A, Taniguchi T (2006) Type I interferon [corrected] gene induction by the interferon regulatory factor family of transcription factors. Immunity 25: 349–360. 16979567 10.1016/j.immuni.2006.08.009

[31] Paun A, Pitha PM (2007) The IRF family, revisited. Biochimie 89: 744–753. 17399883 10.1016/j.biochi.2007.01.014 PMC2139905

[32] Schoggins JW, Wilson SJ, Panis M, Murphy MY, Jones CT, et al (2011) A diverse range of gene products are effectors of the type I interferon antiviral response. Nature 472: 481–485. 10.1038/nature09907 21478870 PMC3409588

[33] Sadler AJ, Williams BR (2008) Interferon-inducible antiviral effectors. Nat Rev Immunol 8: 559–568. 10.1038/nri2314 18575461 PMC2522268

[34] Kotenko SV, Gallagher G, Baurin VV, Lewis-Antes A, Shen M, et al (2003) IFN-lambdas mediate antiviral protection through a distinct class II cytokine receptor complex. Nat Immunol 4: 69–77. 12483210 10.1038/ni875

[35] Verhelst J, Parthoens E, Schepens B, Fiers W, Saelens X (2012) Interferon-inducible protein Mx1 inhibits influenza virus by interfering with functional viral ribonucleoprotein complex assembly. J Virol 86: 13445–13455. 10.1128/JVI.01682-12 23015724 PMC3503048

[36] Hsiang TY, Zhao C, Krug RM (2009) Interferon-induced ISG15 conjugation inhibits influenza A virus gene expression and replication in human cells. J Virol 83: 5971–5977. 10.1128/JVI.01667-08 19357168 PMC2687383

[37] Kim MJ, Hwang SY, Imaizumi T, Yoo JY (2008) Negative feedback regulation of RIG-I-mediated antiviral signaling by interferon-induced ISG15 conjugation. J Virol 82: 1474–1483. 18057259 10.1128/JVI.01650-07 PMC2224411

[38] Malakhova OA, Yan M, Malakhov MP, Yuan Y, Ritchie KJ, et al (2003) Protein ISGylation modulates the JAK-STAT signaling pathway. Genes Dev 17: 455–460. 12600939 10.1101/gad.1056303 PMC195994

[39] Shi HX, Yang K, Liu X, Liu XY, Wei B, et al (2010) Positive regulation of interferon regulatory factor 3 activation by Herc5 via ISG15 modification. Mol Cell Biol 30: 2424–2436. 10.1128/MCB.01466-09 20308324 PMC2863703

[40] Saitoh T, Tun-Kyi A, Ryo A, Yamamoto M, Finn G, et al (2006) Negative regulation of interferon-regulatory factor 3-dependent innate antiviral response by the prolyl isomerase Pin1. Nat Immunol 7: 598–605. 16699525 10.1038/ni1347

[41] Litvak V, Ratushny AV, Lampano AE, Schmitz F, Huang AC, et al (2012) A FOXO3-IRF7 gene regulatory circuit limits inflammatory sequelae of antiviral responses. Nature 490: 421–425. 10.1038/nature11428 22982991 PMC3556990

[42] Driessler F, Venstrom K, Sabat R, Asadullah K, Schottelius AJ (2004) Molecular mechanisms of interleukin-10-mediated inhibition of NF-kappaB activity: a role for p50. Clin Exp Immunol 135: 64–73. 14678266 10.1111/j.1365-2249.2004.02342.x PMC1808913

[43] Sharif MN, Sosic D, Rothlin CV, Kelly E, Lemke G, et al (2006) Twist mediates suppression of inflammation by type I IFNs and Axl. J Exp Med 203: 1891–1901. 16831897 10.1084/jem.20051725 PMC2118370

[44] Sauer I, Schaljo B, Vogl C, Gattermeier I, Kolbe T, et al (2006) Interferons limit inflammatory responses by induction of tristetraprolin. Blood 107: 4790–4797. 16514065 10.1182/blood-2005-07-3058 PMC3963709

[45] Lin KL, Suzuki Y, Nakano H, Ramsburg E, Gunn MD (2008) CCR2+ monocyte-derived dendritic cells and exudate macrophages produce influenza-induced pulmonary immune pathology and mortality. J Immunol 180: 2562–2572. 18250467 10.4049/jimmunol.180.4.2562

[46] Cheung CY, Poon LL, Lau AS, Luk W, Lau YL, et al (2002) Induction of proinflammatory cytokines in human macrophages by influenza A (H5N1) viruses: a mechanism for the unusual severity of human disease? Lancet 360: 1831–1837. 12480361 10.1016/s0140-6736(02)11772-7

[47] Palucka AK, Blanck JP, Bennett L, Pascual V, Banchereau J (2005) Cross-regulation of TNF and IFN-alpha in autoimmune diseases. Proc Natl Acad Sci U S A 102: 3372–3377. 15728381 10.1073/pnas.0408506102 PMC552921

[48] Ivashkiv LB (2003) Type I interferon modulation of cellular responses to cytokines and infectious pathogens: potential role in SLE pathogenesis. Autoimmunity 36: 473–479. 14984024 10.1080/08916930310001605882

[49] Huffman JA, Hull WM, Dranoff G, Mulligan RC, Whitsett JA (1996) Pulmonary epithelial cell expression of GM-CSF corrects the alveolar proteinosis in GM-CSF-deficient mice. J Clin Invest 97: 649–655. 8609219 10.1172/JCI118461 PMC507100

[50] Huang FF, Barnes PF, Feng Y, Donis R, Chroneos ZC, et al (2011) GM-CSF in the lung protects against lethal influenza infection. Am J Respir Crit Care Med 184: 259–268. 10.1164/rccm.201012-2036OC 21474645 PMC6938174

[51] Ghoneim HE, Thomas PG, McCullers JA (2013) Depletion of alveolar macrophages during influenza infection facilitates bacterial superinfections. J Immunol 191: 1250–1259. 10.4049/jimmunol.1300014 23804714 PMC4907362

[52] Hawgood S, Akiyama J, Brown C, Allen L, Li G, et al (2001) GM-CSF mediates alveolar macrophage proliferation and type II cell hypertrophy in SP-D gene-targeted mice. Am J Physiol Lung Cell Mol Physiol 280: L1148–1156. 11350793 10.1152/ajplung.2001.280.6.L1148

[53] Landsman L, Varol C, Jung S (2007) Distinct differentiation potential of blood monocyte subsets in the lung. J Immunol 178: 2000–2007. 17277103 10.4049/jimmunol.178.4.2000

[54] Shi C, Pamer EG (2011) Monocyte recruitment during infection and inflammation. Nat Rev Immunol 11: 762–774. 10.1038/nri3070 21984070 PMC3947780

[55] Wareing MD, Lyon A, Inglis C, Giannoni F, Charo I, et al (2007) Chemokine regulation of the inflammatory response to a low-dose influenza infection in CCR2-/- mice. J Leukoc Biol 81: 793–801. 17179466 10.1189/jlb.0506299

[56] Dawson TC, Beck MA, Kuziel WA, Henderson F, Maeda N (2000) Contrasting effects of CCR5 and CCR2 deficiency in the pulmonary inflammatory response to influenza A virus. Am J Pathol 156: 1951–1959. 10854218 10.1016/S0002-9440(10)65068-7 PMC1850091

[57] Taut K, Winter C, Briles DE, Paton JC, Christman JW, et al (2008) Macrophage Turnover Kinetics in the Lungs of Mice Infected with Streptococcus pneumoniae. Am J Respir Cell Mol Biol 38: 105–113. 17690327 10.1165/rcmb.2007-0132OC

[58] Seo SU, Kwon HJ, Ko HJ, Byun YH, Seong BL, et al (2011) Type I interferon signaling regulates Ly6C(hi) monocytes and neutrophils during acute viral pneumonia in mice. PLoS Pathog 7: e1001304 10.1371/journal.ppat.1001304 21383977 PMC3044702

[59] Schliehe C, Flynn EK, Vilagos B, Richson U, Swaminathan S, et al (2014) The methyltransferase Setdb2 mediates virus-induced susceptibility to bacterial superinfection. Nat Immunol. 10.1038/ni.3046 PMC4320687 25419628

[60] Sun J, Madan R, Karp CL, Braciale TJ (2009) Effector T cells control lung inflammation during acute influenza virus infection by producing IL-10. Nat Med 15: 277–284. 10.1038/nm.1929 19234462 PMC2693210

[61] Brown DM, Lee S, Garcia-Hernandez Mde L, Swain SL (2012) Multifunctional CD4 cells expressing gamma interferon and perforin mediate protection against lethal influenza virus infection. J Virol 86: 6792–6803. 10.1128/JVI.07172-11 22491469 PMC3393557

[62] Bacon KB, Premack BA, Gardner P, Schall TJ (1995) Activation of dual T cell signaling pathways by the chemokine RANTES. Science 269: 1727–1730. 7569902 10.1126/science.7569902

[63] Havenar-Daughton C, Kolumam GA, Murali-Krishna K (2006) Cutting Edge: The direct action of type I IFN on CD4 T cells is critical for sustaining clonal expansion in response to a viral but not a bacterial infection. J Immunol 176: 3315–3319. 16517698 10.4049/jimmunol.176.6.3315

[64] Lillard JW Jr., Boyaka PN, Taub DD, McGhee JR (2001) RANTES potentiates antigen-specific mucosal immune responses. J Immunol 166: 162–169. 11123289 10.4049/jimmunol.166.1.162

[65] Lee JK, Kim JK, Lee YR, Kim HS, Im SA, et al (2005) Exposure to chemokines during maturation modulates antigen presenting cell function of mature macrophages. Cell Immunol 234: 1–8. 15950960 10.1016/j.cellimm.2005.04.017

[66] Montoya M, Schiavoni G, Mattei F, Gresser I, Belardelli F, et al (2002) Type I interferons produced by dendritic cells promote their phenotypic and functional activation. Blood 99: 3263–3271. 11964292 10.1182/blood.v99.9.3263

[67] Moyron-Quiroz JE, Rangel-Moreno J, Kusser K, Hartson L, Sprague F, et al (2004) Role of inducible bronchus associated lymphoid tissue (iBALT) in respiratory immunity. Nat Med 10: 927–934. 15311275 10.1038/nm1091

[68] Marazzi I, Ho JS, Kim J, Manicassamy B, Dewell S, et al (2012) Suppression of the antiviral response by an influenza histone mimic. Nature 483: 428–433. 10.1038/nature10892 22419161 PMC3598589

[69] Ehrhardt C, Wolff T, Pleschka S, Planz O, Beermann W, et al (2007) Influenza A virus NS1 protein activates the PI3K/Akt pathway to mediate antiapoptotic signaling responses. J Virol 81: 3058–3067. 17229704 10.1128/JVI.02082-06 PMC1866065

[70] Talon J, Horvath CM, Polley R, Basler CF, Muster T, et al (2000) Activation of interferon regulatory factor 3 is inhibited by the influenza A virus NS1 protein. J Virol 74: 7989–7996. 10933707 10.1128/jvi.74.17.7989-7996.2000 PMC112330

[71] Coulombe F, Jaworska J, Verway M, Tzelepis F, Massoud A, et al (2014) Targeted prostaglandin E2 inhibition enhances antiviral immunity through induction of type I interferon and apoptosis in macrophages. Immunity 40: 554–568. 10.1016/j.immuni.2014.02.013 24726877

[72] Hamilton BE, Minino AM, Martin JA, Kochanek KD, Strobino DM, et al (2007) Annual summary of vital statistics: 2005. Pediatrics 119: 345–360. 17272625 10.1542/peds.2006-3226

[73] Shahangian A, Chow EK, Tian X, Kang JR, Ghaffari A, et al (2009) Type I IFNs mediate development of postinfluenza bacterial pneumonia in mice. J Clin Invest 119: 1910–1920. 10.1172/JCI35412 19487810 PMC2701856

[74] Li W, Moltedo B, Moran TM (2012) Type I interferon induction during influenza virus infection increases susceptibility to secondary Streptococcus pneumoniae infection by negative regulation of gammadelta T cells. J Virol 86: 12304–12312. 10.1128/JVI.01269-12 22951826 PMC3486468

[75] Nakamura S, Davis KM, Weiser JN (2011) Synergistic stimulation of type I interferons during influenza virus coinfection promotes Streptococcus pneumoniae colonization in mice. J Clin Invest 121: 3657–3665. 10.1172/JCI57762 21841308 PMC3163966

[76] Pettitt SJ, Liang Q, Rairdan XY, Moran JL, Prosser HM, et al (2009) Agouti C57BL/6N embryonic stem cells for mouse genetic resources. Nat Methods 6: 493–495. 10.1038/nmeth.1342 19525957 PMC3555078

[77] Ryder E, Gleeson D, Sethi D, Vyas S, Miklejewska E, et al (2013) Molecular characterization of mutant mouse strains generated from the EUCOMM/KOMP-CSD ES cell resource. Mamm Genome 24: 286–294. 10.1007/s00335-013-9467-x 23912999 PMC3745610

[78] Kranz A, Fu J, Duerschke K, Weidlich S, Naumann R, et al (2010) An improved Flp deleter mouse in C57Bl/6 based on Flpo recombinase. Genesis 48: 512–520. 10.1002/dvg.20641 20506501

[79] Clausen BE, Burkhardt C, Reith W, Renkawitz R, Forster I (1999) Conditional gene targeting in macrophages and granulocytes using LysMcre mice. Transgenic Res 8: 265–277. 10621974 10.1023/a:1008942828960

[80] Ito T, Schaller M, Hogaboam CM, Standiford TJ, Sandor M, et al (2009) TLR9 regulates the mycobacteria-elicited pulmonary granulomatous immune response in mice through DC-derived Notch ligand delta-like 4. J Clin Invest 119: 33–46. 10.1172/JCI35647 19075396 PMC2613456

[81] Reed M, Morris SH, Jang S, Mukherjee S, Yue Z, et al (2013) Autophagy-inducing protein beclin-1 in dendritic cells regulates CD4 T cell responses and disease severity during respiratory syncytial virus infection. J Immunol 191: 2526–2537. 10.4049/jimmunol.1300477 23894198 PMC3811020

