# Supplementary material for: Type I Interferon Induced Epigenetic Regulation of Macrophages Suppresses Innate and Adaptive Immunity in Acute Respiratory Viral Infection
Source: PLoS Pathog. 2015 Dec 28;11(12):e1005338. doi: 10.1371/journal.ppat.1005338 (PMC4692439; doi:10.1371/journal.ppat.1005338)
Supplement: S1 Table — (PDF) [file ppat.1005338.s001.pdf]

S1 Table. Genotyping primers and PCR conditions

Gene Trap Mice

Primer	Sequence
KOMP universal	CACAACGGGTTCTTCTGTTAGTCC
Gene Trap	GAATCAAGACTAGAAGGCGACTACTG

Floxed Mice

Primer	Sequence
Setdb2 3' universal	CACAACGGGTTCTTCTGTTAGTCC
Setdb2 wild-type	GAATCAAGACTAGAAGGCGACTACTG
KOMP allele	TCTATAGTCGCAGTAGGCGG

PCR conditions

Cycles	Temperature	Time
1	95°C	2 min
25	95°C	30 sec
	57°C	40 sec
	72°C	3 min
1	72°C	7 min

PCR products

Primers	Product
Setdb2 3' universal + wild-type	250 basepairs
Setdb2 3' universal + KOMP	900 basepairs
