# Supplementary material for: Type I Interferon Induced Epigenetic Regulation of Macrophages Suppresses Innate and Adaptive Immunity in Acute Respiratory Viral Infection
Source: PLoS Pathog. 2015 Dec 28;11(12):e1005338. doi: 10.1371/journal.ppat.1005338 (PMC4692439; doi:10.1371/journal.ppat.1005338)
Supplement: S2 Table — (PDF) [file ppat.1005338.s002.pdf]

S2 Table. Custom ChIP primers

Promotor	Sequence
Isg15	F: CTGCCGCCCGCCCTCTCC<br>R: CCCCGCCCCGCCCACTCTA
Mx1	F: TGGGCACAGACAACCTTA<br>R:TCCTCAGAGCCCTTAGACT
Ccl2	F: CAACAAGGCCTGATAACCA<br>R: GGAAAGGACCCCCAGTAAG

***Setdb2* Promoter**	**Sequence**
STAT1 binding site 1	F: TTTGAAACTGGGGTAAGGGTGTG<br>R: ATAAAAAGGAGAAACGGGAAGTAA
STAT1 binding site 2	F: AGTCGCGTTTCCCCCACCGTCTTG<br>R: TCTTGTCTGTGCTCCGCTGTCTAC
IRF7 binding site 1	F: CTCAATTTTCACATGCTCTAACCT<br>R: ACAGTCTAAATTCCTCCTTGCTAT
IRF7 binding site 2	F: TTTGAAACTGGGGTAAGGGTGTG<br>R: ATAAAAAGGAGAAACGGGAAGTAA
